# Class II LitR serves as an effector of “short” LOV-type blue-light photoreceptor in *Pseudomonas mendocina*

**DOI:** 10.1038/s41598-022-26254-3

**Published:** 2022-12-16

**Authors:** Takafumi Maruyama, Satoru Sumi, Mitsuru Kobayashi, Teppei Ebuchi, Yu Kanesaki, Hirofumi Yoshikawa, Kenji Ueda, Hideaki Takano

**Affiliations:** 1 Life Science Research Center, College of Bioresource Sciences, Nihon University, 1866 Kameino, Fujisawa, Kanagawa 252-0880 Japan; 2 Genome Research Center, Tokyo University of Agriculture, 1-1-1 Sakuragaoka, Tokyo, Setagaya-ku 156-8502 Japan; 3 Department of Bioscience, Tokyo University of Agriculture, 1-1-1 Sakuragaoka, Tokyo, Setagaya-ku 156-8502 Japan; 4 Present Address: Research Institute of Green Science and Technology, Shizuoka University, 836 Ohya, Suruga-ku, Shizuoka 422-8529 Japan

**Keywords:** Molecular biology, Transcription, Transcriptional regulatory elements

## Abstract

PmlR2, a class II LitR/CarH family transcriptional regulator, and PmSB-LOV, a “short” LOV-type blue light photoreceptor, are adjacently encoded in *Pseudomonas mendocina* NBRC 14162. An effector protein for the “short” LOV-type photoreceptor in *Pseudomonas* has not yet been identified. Here, we show that PmlR2 is an effector protein of PmSB-LOV. Transcriptional analyses revealed that the expression of genes located near *pmlR2* and its homolog gene, *pmlR1,* was induced in response to illumination. In vitro DNA–protein binding analyses showed that recombinant PmlR2 directly binds to the promoter region of light-inducible genes. Furthermore PmSB-LOV exhibited a typical LOV-type light-induced spectral change. Gel-filtration chromatography demonstrated that the illuminated PmSB-LOV was directly associated with PmlR2, whereas non-illuminated proteins did not interact. The inhibition of PmlR2 function following PmSB-LOV binding was verified by surface plasmon resonance: the DNA-binding ability of PmlR2 was specifically inhibited in the presence of blue light-illuminated-PmSB-LOV. An In vitro transcription assay showed a dose-dependent reduction in PmlR2 repressor activity in the presence of illuminated PmSB-LOV. Overall, evidence suggests that the DNA-binding activity of PmlR2 is inhibited by its direct association with blue light-activated PmSB-LOV, enabling transcription of light-inducible promoters by RNA polymerase.

## Introduction

The LitR (light-induced transcription; regulator)/CarH protein family are a MerR-type transcriptional regulator containing C-terminal vitamin B_12_-binding domains. LitR/CarH plays a central role in light-inducible carotenoid production in several bacteria including *Myxococcus xanthus*^1^; a gram-negative gliding bacterium*, Streptomyces coelicolor* A3(2)^2^; the gram-positive antibiotic producer, *Bacillus megaterium* QM B1551^3^; a low-GC endospore-forming gram-positive bacterium; and *Thermus thermophilus* HB27^4^, a thermophilic gram-negative bacterium. Previously, we revealed the molecular mechanism underlying LitR-mediated light-inducible transcription in *B. megaterium* QM B1551^3^. The LitR of *B. megaterium* associates with adenosyl B_12_ (AdoB_12_) and serves as a negative transcriptional regulator. Upon illumination, the LitR protein is inactivated by the photolysis of AdoB_12_, due to the light sensitivity of the Co–C bond, preventing DNA-binding activity^3^. A similar light-response mechanism involving LitR has been reported in *T. thermophilus*^1^ and *Myxococcus xanthus*^5^. These studies suggest that members of the LitR/CarH family serve as light-dependent regulators in phylogenetically diverse non-phototrophic bacteria and are mainly involved in the light-inducible carotenoid production^1,6,7^.

The completion of multiple genome sequences has revealed a wide distribution of LitR homologs in divergent bacterial genomes, including gram-positive and gram-negative bacteria^8^. Recently, we reported that there are at least five classes of LitR/CarH family proteins (designated class I to V LitR) based on phylogenetic analysis of the amino acid sequences of the C-terminal domains^8^, which have diverged, in contrast to the N-terminal HTH DNA-binding domains. Generally, MerR-type regulators possess an HTH-DNA-binding domain at the N-terminus and an effector-binding domain at the C-terminus. As described above, class I LitRs are AdoB_12_-based photosensory regulators. Class II LitRs are mainly found within the genus *Pseudomonas* and retain only the 4-helix bundle domain, one of two B_12_-binding domains, implying that they have no ability to associate with B_12_^9^. Class III, IV, and V LitRs are mainly distributed in the genera *Burkholderia*^8^, *Vibrio*, and *Micrococcales*, respectively. Although the C-termini of class III LitRs shows no similarity to the functional domain, analysis of the recombinant protein showed a light-dependent change in the absorption spectrum, suggesting that the class III LitR possesses a novel light-sensing mechanism^8^. Class IV and V LitRs do not possess a B_12_-binding domain, and their functions are unknown. These results suggest that LitR/CarH family proteins in non-phototrophic bacteria have the ability to sense and respond to light with diverse light-sensing mechanisms.

Light, oxygen, or voltage (LOV) proteins contain a flavin mononucleotide (FMN) chromophore and play an important role in the regulation of multiple blue-light dependent cellular functions in plants, fungi, and bacteria^10^. Bacterial LOV proteins are mainly classified into two types based on the presence or absence of an effector domain, which transmits the light signal to other domains or proteins, such as a transcriptional regulators or kinases^11^. The relationship between the photoreceptor domain and the effector domain has been studied in LOV proteins retaining the effector domains^12–15^. LOV proteins lacking effector domains are known as “short” LOVs. The photochemistry of three “short” bacterial LOVs has also been previously studied: PpSB1-LOV^16–18^ and PpSB2-LOV^18,19^ of *P. putida* KT2440, RsLOV of *Rhodobacter sphaeroides*, a purple photosynthetic gram-negative bacterium^20^, and DsLOV of *Dinoroseobacter shibae*, a marine phototrophic bacterium^21^. However, no binding partners of these “short” LOVs have yet been identified.

*Pseudomonas putida* KT2440, a gram-negative soil bacterium, harbors multiple LitR homologs (PplR1-PplR3) designated as class II LitR^9^. We recently reported that three *pplR* genes are cooperatively involved in the expression of 19 light-inducible genes including those encoding PplR1*,* PhrB (encoding DNA photolyase), UfaM (furan-containing fatty acid synthase), FolE (GTP cyclohydrolase I), CryB (cryptochrome-like protein), and multiple genes without annotated/known functions^9^. Knockout of *ppSB1-LOV* and *ppSB2-LOV*, homologs of the plant-specific blue-light photoreceptor phototropin, caused constitutive low-level transcription of PplR-dependent genes under both dark and light conditions, suggesting that PpSB-LOVs control PplR activity via an unknown mechanism^9^.

Our previous study suggested that *ppSB-LOV* genes control PplR-dependent light-inducible transcription^9^; however, in vitro analyses of this mechanism faced limitations. For example, reconstructing protein–protein interactions are hampered by the difficulty of preparing a soluble and tag-free PplR recombinant protein. However, we anticipated that further elucidation of a photo-regulatory mechanism mediated by a “short” LOV protein and a MerR family protein would provide new insights into the mechanism of the bacterial photo-response. Here, we found that recombinant proteins derived from *Pseudomonas mendocina* NBRC 14162 can be stably purified as soluble proteins, and investigated the function of PmlR2 (*Pseudomonas mendocina* light-inducible transcriptional regulator), a homolog of PplR, and PmSB-LOV (*Pseudomonas mendocina* Sensory box-LOV), a homolog of PpSB-LOV. This study indicated that the DNA-binding activity of PmlR2 is negatively controlled by PmSB-LOV via light-dependent protein–protein interactions. We provide the first biochemical evidence that a MerR-type transcriptional regulator is an effector protein of a “short” LOV type blue light photoreceptor in bacteria.

## Results

### *pmlR* and *pmSB-LOV* genes of Pseudomonas mendocina NBRC 14162

**Figure 1:**
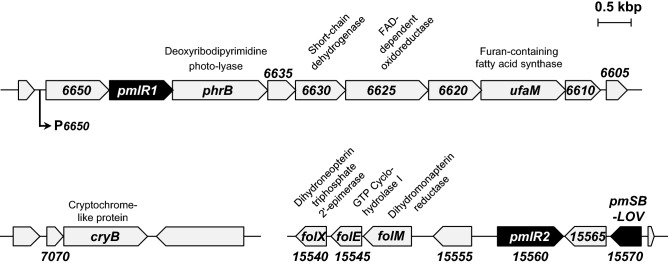
Gene organization of the *pmlR1* and *PmlR2* genomic regions. P*6650* is a light-inducible promoter analyzed in this study. The homologous genes with known functions include *phrB*, a deoxyribodipyrimidine photo-lyase; *PME6630*, a short-chain dehydrogenase; *PME6625*, a FAD-dependent oxidoreductase; *ufaM*, a furan-containing fatty acid synthase; *cryB*, a cryptochrome/photolyase family protein; *folX*, a dihydroneopterin triphosphate 2′-epimerase; *folE*, a GTP cyclohydrolase I; and *folM*, a dihydromonapterin reductase. Other *ORFs* encode proteins without annotated/known function.

*P. mendocina* NBRC 14162 is a gram-negative bacterium isolated by soil enriched with ethanol as a carbon source^22^. Figure 1 shows a schematic representation of the genomic organization of two *pplR* homologs (designated *pmlR1* and *pmlR2*) and a homolog of *ppSB-LOV* (designated *pmSB-LOV*) in *P. mendocina* NBRC14162.

The *pmlR1* region contains genes encoding a deoxyribodipyrimidine photo-lyase (PhrB), short-chain dehydrogenase (PME6630), FAD-dependent oxidoreductase (PME6625), furan-containing fatty acid synthase (UfaM), and hypothetical proteins (PME6650, PME6635, PME6620, and PME6610). The *pmlR1* region is highly conserved among *Pseudomonas spp.* and other closely related bacteria^9^. The *pmlR2* region and *pmlR1* cluster in this bacterium are encoded at different loci, similar to genome-sequenced *P. mendocina* NK-01^23^ and ymp^24^. The *pmlR2* region includes genes encoding PmSB-LOV, Dihydromonapterin reductase (FolM), GTP Cyclohydrolase I (FolE), Dihydroneopterin triphosphate 2′-epimerase (FolX), and a hypothetical protein (PME15555). FolM, FolE, and FolX likely comprise an operon involved in folate and pterin biosynthesis^25^. The gene cluster, comprising *pmlR2*, *PME15565*, and *pmSB-LOV* genes, was conserved in the chromosome of *Pseudomonas* spp.

The *pmlR2* gene encodes a protein of 321 amino acids (calculated molecular weight: 35.82 kDa) and shares 56%, 39%, and 34% identity with PplR3, PplR2, and PplR1 of *P. putida*, respectively (Fig. S1A). In contrast, PmlR1 (318 aa, 35.56 kDa) shares 45%, 36%, and 28% amino acid identity with PplR1, PplR3, and PplR2, respectively. PmlR2 shares a low identity (26.4%) with PmlR1 across the entire sequence, but its N-terminal MerR-type HTH DNA-binding domain shares 70% similarity. This high similarity in the HTH domain was also observed for PplR proteins (Fig. S1A). A Pfam search showed that the internal regions of PmlR1 and PmlR2, residues Arg^106^ to Arg^179^ (PmlR1) and Trp^113^ to Leu^161^ (PmlR2), share similarity with a B_12_-binding_2 domain (PF02607) with *E*-values of 1.1e-05 and 3.5e-07, respectively. The C-termini of both PmlR proteins shared no similarity with known functional protein domains. Generally, two domains, the B_12_-binding domain and the B_12_-binding_2 domain, are required for association with B_12_, suggesting that PmlRs have no ability to form a complex with B_12_.

PmSB-LOV consists of 148 amino acids (16.68 kDa), including a typical “short” LOV protein lacking an effector domain. PmSB-LOV shares 100% identity with MDS_3586 of *P. mendocina* NK-01 (Fig. S1B), and end-to-end similarity with PpSB2-LOV (71%) and PpSB1-LOV (69%) of *P. putida* KT2440. A conserved cysteine residue required for the thiol-adduct formation with FMN was also present at the position 53 (designated as Cys^53^) of the PmSB-LOV protein. The conserved N-terminal N-cap and C-terminal Jα-helix found in other “short” LOVs was also found in PmSB-LOV (Fig. S1B).

We focused on PmlR2 and PmSB-LOV because of their proximity in the genome of *P. mendocina* NBRC 14162 and likely strong functional relationship.

### Light-inducible transcription in *P. mendocina* NBRC 14162

We performed RNA-seq analysis to preliminary screen genes whose expression is changed in response to blue light. Total RNA was purified from cells cultured in liquid medium under dark and blue light conditions at an intensity of 30.5 µmol s^−1^ m^−2^. Light-induced genes more than two-fold are summarized in Table S1. The identified light-inducible genes were common to *P. putida* KT2440, as previously reported by us^9^.

To confirm the results of RNA-seq analysis, we performed quantitative RT-PCR (qRT-PCR). The transcription of *pmlR1*, *phrB*, and *ufaM* was much higher under light conditions (Table 1). Transcription of *pmlR2* and *pmSB-LOV* was constitutive under dark and light conditions. Thus, many of the genes located in the flanking region of *pmlR1* were strongly transcribed in a photo-dependent manner, and those of *pmlR2* were slightly upregulated in light conditions. *phrB* and *ufaM* in *P. putida* KT2440 are similarly regulated by PplRs, suggesting that these genes are regulated by PmlR1 and PmlR2 in this bacterium.

**Table 1:** Light-induced fold change of transcription^a^.

Gene	Cultivation time		
	6 h	14 h	21 h
*pmlR1*	10.1 ± 0.4	6.0 ± 0.3	5.7 ± 1.1
*phrB*	27.8 ± 1.6	33.2 ± 14.1	26.7 ± 5.3
*ufaM*	25.0 ± 0.8	33.2 ± 1.8	32.2 ± 6.3
*pmSB-LOV*	1.1 ± 0.0	1.0 ± 0.0	0.9 ± 0.2
*pmlR2*	1.5 ± 0.0	1.5 ± 0.1	1.4 ± 0.3
*folM*	2.4 ± 0.1	2.5 ± 0.1	2.5 ± 0.5
*folE*	1.6 ± 0.0	3.1 ± 0.1	3.3 ± 0.6
*PME_16455*	1.2 ± 0.0	1.8 ± 0.1	1.2 ± 0.2
*PME_16460*	1.2 ± 0.0	1.3 ± 0.1	1.2 ± 0.2
*PME_21940*	1.3 ± 0.0	1.1 ± 0.1	1.8 ± 0.4
*PME_21945*	1.1 ± 0.1	1.2 ± 0.1	1.6 ± 0.3
*rpoD*	1.1 ± 0.0	0.8 ± 0.0	1.1 ± 0.2

^a^Intensity of the strain cultured under the light condition normalized to that of the strain cultured under the dark condition.

We predicted the transcriptional units based on the results of RNA-seq and qRT-PCR analyses. Nine genes (*PME6650* to *PME6610*), two genes (*PME7070* and *cryB*)*,* and three genes (*folM*, *folE*, and *folX*) likely comprise a polycistron, transcribed by the promoter preceding the first gene, as their transcriptional levels detected by RNA-seq analysis showed a similar fold change in response to illumination (Table S1). The putative translational start codon for each downstream coding sequence from the first gene of the operon overlapped or was close with the stop codon of the preceding coding sequence.

It is important to understand the in vivo function of the *pmlR* and *pmSB-LOV* genes in *P. mendocina* NBRC 14162. However, we were unable to establish a transformation method by conjugative transfer or electroporation using several types of vectors, including a broad-host-range plasmid (pBHR1) and genome-integrative transposon (pUTminiTn5). Thus, we mainly focused on the in vitro analysis of PmlR2 and PmSB-LOV.

### Promoter structure preceding the light-inducible *PME6650* gene

**Figure 2:**
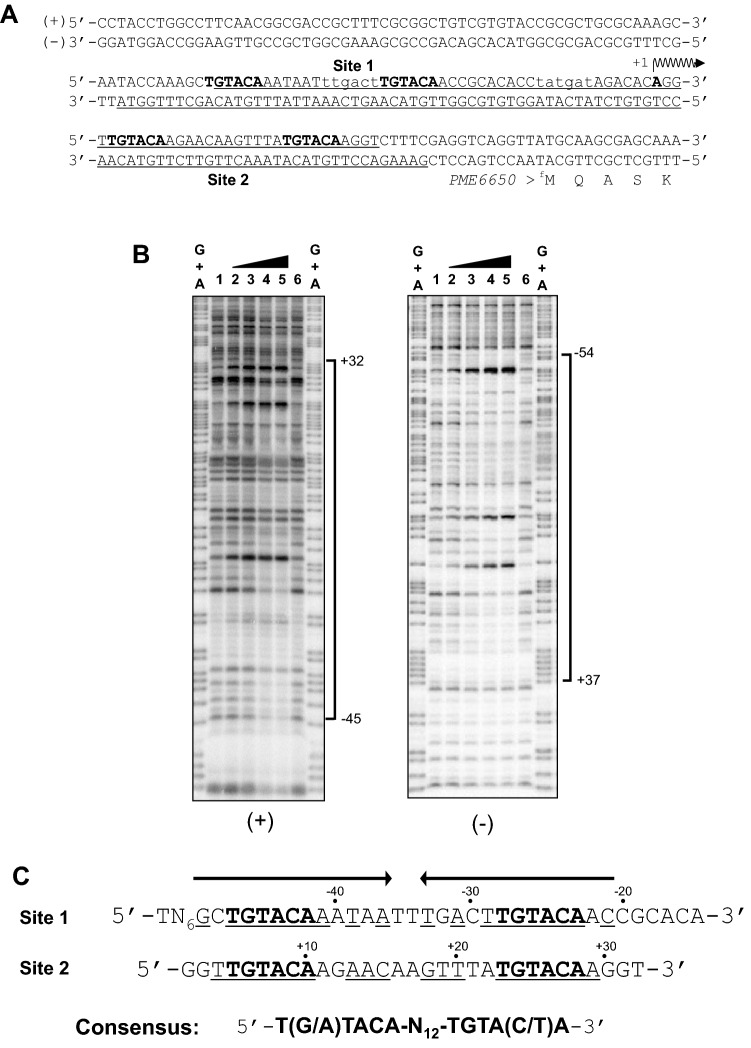
Determination of the PmlR2-binding sites by DNase I footprint analysis. (**A**) The nucleotide sequence of P*6650*. PmlR2-binding sites are underlined. The consensus (5′-T(G/A)TACA and TGTACA) for *P. putida* PplR1-binding is denoted by bold letters including Site 1 and Site 2. The transcriptional start site of P*6650*, as determined by 5′-RACE and CAGE, is indicated by + 1 and a vent arrow. Putative − 10 and − 35 hexamer sequences are shown in small letters. (**B**) DNase I footprint assay. One PmlR2-binding site in the sense ( +) and antisense ( −) strands of P*6650* was protected. The amounts of recombinant PmlR2 used were 0 pmol (lane 1 and 6), 10 pmol (lane 2), 20 pmol (lane 3), 40 pmol (lane 4), and 80 pmol (lane 5), which were added to the reaction mixture with 10 kcpm ^32^P-labeled DNA probes. Three independent experiments in technical replicates were performed, and representative data were shown. The original and unprocessed versions of the full-length gels were included in Figs. S10 and S11. (**C**) The alignment of the two PmlR2-binding-sites with the *P. putida* PplR1 consensus.

To assign the transcriptional start site of the *PME6650* promoter (designated P*6650*) of *P. mendocina* NBRC 14162 and *PP0468* (designated P*468*) of *P. putida* KT2440 used for the control promoter in vitro transcriptional runoff assay as described below, we performed 5′-RACE and CAGE experiments. The transcriptional start sites of P*6650* and P*468* were located 48 bp and 51 bp upstream of the ATG translation initiation codon (Fig. 2A). The −35 and −10 regions of P*6650* (TTGACT_N16_TATGAT) were highly similar to the consensus sequence of the *Escherichia coli* σ^70^-dependent promoter (TTGACA_N16–18_TATAAT)^26^. Based on the position of the transcriptional start site, we annotated a different translational initiation codon for *PME6650* in *P. mendocina* NBRC 14162 than that in *P. mendocina* NK-01.

We first evaluated the DNA-binding activity of the recombinant PmlR2 protein using a gel-shift assay. PmlR2 was expressed as a Glutathione-S-Transferase (GST)-fusion protein in *E. coli* and purified using GST affinity chromatography. The GST-tag was removed using a sequence-specific protease (See Materials and Methods), and the tag-free PmlR2 recombinant protein was used for the gel-shift assay and other experiments. PmlR2 bound to the promoter region of *PME6650* and *folM* but did not bind to the *rpoD* promoter region at low protein concentrations (Fig. S2). Among these promoter regions, the most stable band shift was observed when P*6650* was used as the probe. Therefore, we used P*6650* to study PmlR2 DNA-binding activity in this study.

DNase I footprint analysis was used to identify the PmlR2-binding sites on the sense and antisense strands of P*6650* (Fig. 2A and B). Single broad regions in the sense strand (+ 32 to − 45) and antisense strand (+ 37 to − 54) were protected from DNase I treatment, indicating that P*6650* contains multiple PmlR2-binding sites (Fig. 2B). The supporting data shown in Fig. S3 was used to analyze the result of the sense strand in Fig. 2B, as the specific DNA-binding activity of PmlR2 with the sense strand was low compared to that of the antisense strand. We compared the nucleotide sequences of the protected regions with the consensus of PplR1^9^. As shown in Fig. 2A and C, the nucleotide sequences 5′-TGTACA-N_12_-TGTACA-3', designated as Site 1, and 5′-TGTACA-N_12_-TGTACA-3', designated as Site 2, shared high similarity with the consensus sequences (5′-T(G/A)TACA-N_12_-TGTA(C/T)A-3') of PplR1^9^. Site 1 overlaps with the  − 35 region of P*6650*, Site 2 is located between the transcriptional start site and the translational initiation codon of *PME6650*.

### Light-dependent change in the absorbance spectrum of PmSB-LOV recombinant protein

The LOV protein generally shows light-dependent absorption changes (called photocycles) in the UV–visible region (300–600 nm) due to the formation of a thiol adduct between the conserved Cys residue of LOV and FMN upon illumination. The LOV domain absorption ability is restored to the dark state in the absence of light (dark state recovery). To investigate the changes in absorption, we purified wild-type PmSB-LOV and PmSB-LOV_C53A_ recombinant proteins from an *E. coli* expression host (See Materials and Methods). Both purified proteins were yellow, consistent with their association with flavin derivatives, characteristic of LOV proteins. HPLC analyses showed that both types of PmSB-LOV proteins were predominately associated with FMN and a smaller amount of FAD chromophore (Fig. S4). This result indicates two things: (i) FMN is a major chromophore of PmSB-LOV, and (ii) the Cys^53^ residue is not involved in the association with flavins.

**Figure 3:**
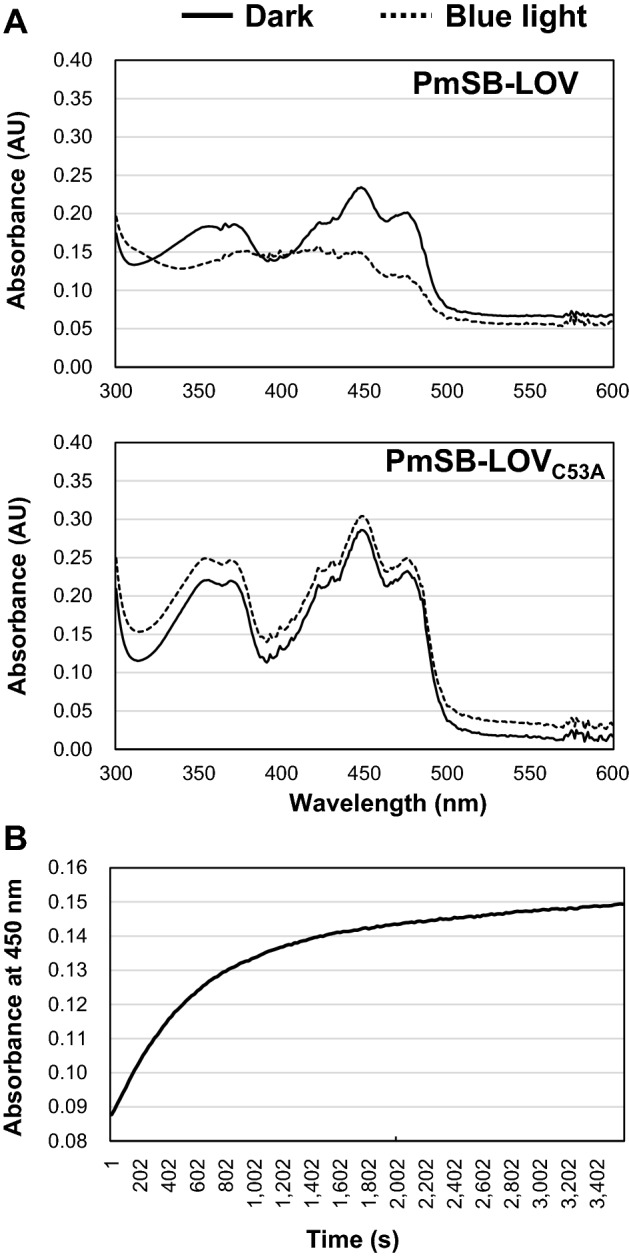
Absorption spectra of PmSB-LOV. (**A**) The absorbance spectra of the dark state and photoequilibrium (10 s of blue-light illumination) of the wild-type PmSB-LOV (upper panel) and PmSB-LOV_C53A_ (lower panel) are shown by the solid and dashed line, respectively. (**B**) Dark state recovery of the wild type PmSB-LOV. Measurements of dark state recovery rates were taken every 20 s for 1 h by tracing absorbance at 450 nm. Two independent experiments in technical replicates were performed, and representative data were shown.

We then measured the absorbance spectrum (300–600 nm) of PmSB-LOV in dark and light conditions at 25 °C. The absorption spectrum of dark-incubated PmSB-LOV contained multiple peaks, similar to a typical LOV profile. The PmSB-LOV spectrum changed upon exposure to 450 nm blue light at 100 μmol s^−1^ m^−2^ for 10 s (Fig. 3A), whereas exposure to light at 365, 530, or 660 nm had no effect. Thus, PmSB-LOV exhibited typical absorbance spectrum behavior LOV proteins.

We also analyzed the spectra of PmSB-LOV_C53A_ to assess the involvement of the conserved Cys^53^ residue in the change in absorbance. Unlike the wild-type protein, PmSB-LOV_C53A_ exhibited similar absorption spectra under both dark and light conditions (Fig. 3A), suggesting that the Cys^53^ residue is required for the formation of the thiol-adduct with FMN, similar to other LOV proteins.

Next, the absorbance was monitored at 450 nm for 1 h to trace the dark state recovery. Full recovery to the dark state was observed after 56 min (Fig. 3B). This indicated that PmSB-LOV is a relatively slow-reverting LOV protein.

### Protein–protein interaction between PmlR2 and PmSB-LOV in heterologous *E. coli*

**Figure 4:**
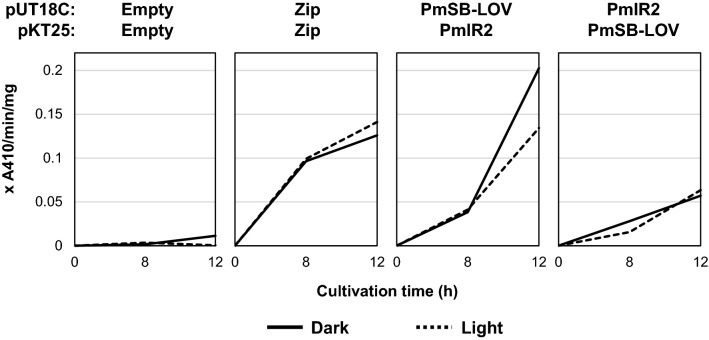
Two-hybrid system analysis of PmlR2 and PmSB-LOV in *E. coli*. The *E. coli* BTH101 transformants carrying pUT18C-*pmSB-LOV*/pKT25-*pmlR2* and pUT18C-*pmlR2*/pKT25-*pmSB-LOV* were cultured under dark (solid line) and light (dashed line) conditions. In each vector, PmlR2 and PmSB-LOV were fused at the C-terminus of the split adenylate cyclase domain. β-galactosidase activity indicating protein–protein interaction was measured at 8 and 12 h. The BTH101 cells carrying pUT18C/pKT25 and pUT18zip/pKT25zip were used as negative and positive controls, respectively. Two independent experiments in biological replicates were performed, and representative data were shown.

To obtain preliminary information on the interaction between PmlR2 and PmSB-LOV in vivo, we performed a two-hybrid analysis using *E. coli* as a heterologous host. In each construct, PmlR2 and PmSB-LOV were fused at the C-terminal domain of each split cAMP synthase domain (T18 and T25). As shown in Fig. 4, *E. coli* transformants carrying *pmlR2* and *ppSB-LOV* exhibited higher β-galactosidase activity than the negative (empty/empty) and positive (zip/zip) control strain. The β-galactosidase activities generated by the interaction were observed under dark and light conditions. We performed the same analysis with *E. coli* transformants carrying *pmlR2* and *ppSB-LOV*, in which both genes were fused with various pairs of cAMP synthase domains (Fig. S5). We assume that chimeric construction via C-terminal or N-terminal fusion with the cAMP synthase domain prevents light-dependent protein–protein interaction.

### Light-dependent complex formation composed of PmlR2 and PmSB-LOV

**Figure 5:**
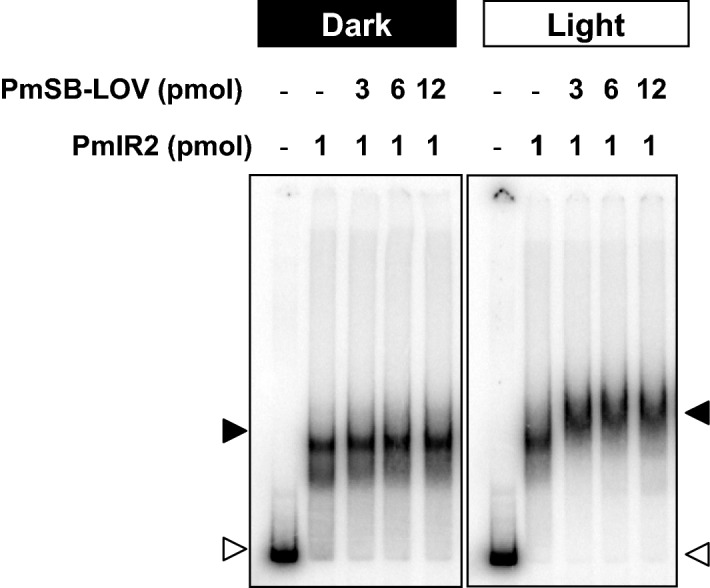
Gel-shift assay of PmlR2 and PmSB-LOV with P*6650*. The indicated amounts of proteins were incubated under dark (left panel) or white light (right panel) conditions before the addition of a total of 0.5–5.0 ng of ^32^P-labeled probe. Continuous irradiation of the polyacrylamide gel during electrophoresis was required for the super shift composed of PmlR2, PmSB-LOV, and the probe. Open and closed triangle denote the probe and protein-DNA complex, respectively. Three independent experiments in technical replicates were performed, and representative data were shown. The original and unprocessed versions of the full-length gels were included in Fig. S12.

We performed a gel-shift assay in the presence of PmSB-LOV to examine the association between PmlR2 and PmSB-LOV. When P*6650* was used as the probe, the addition of non-illuminated PmSB-LOV caused a partial super shift (Fig. 5). On the other hands, the addition of white light-treated PmSB-LOV caused a clear super shift, and a partial dose-dependent super-shift was observed (Fig. 5). This strongly suggests that illuminated PmSB-LOV directly interacts with PmlR2, while non-illuminated PmSB-LOV has the low activity.

### Light-dependent protein–protein interaction between PmlR2 and PmSB-LOV

**Figure 6:**
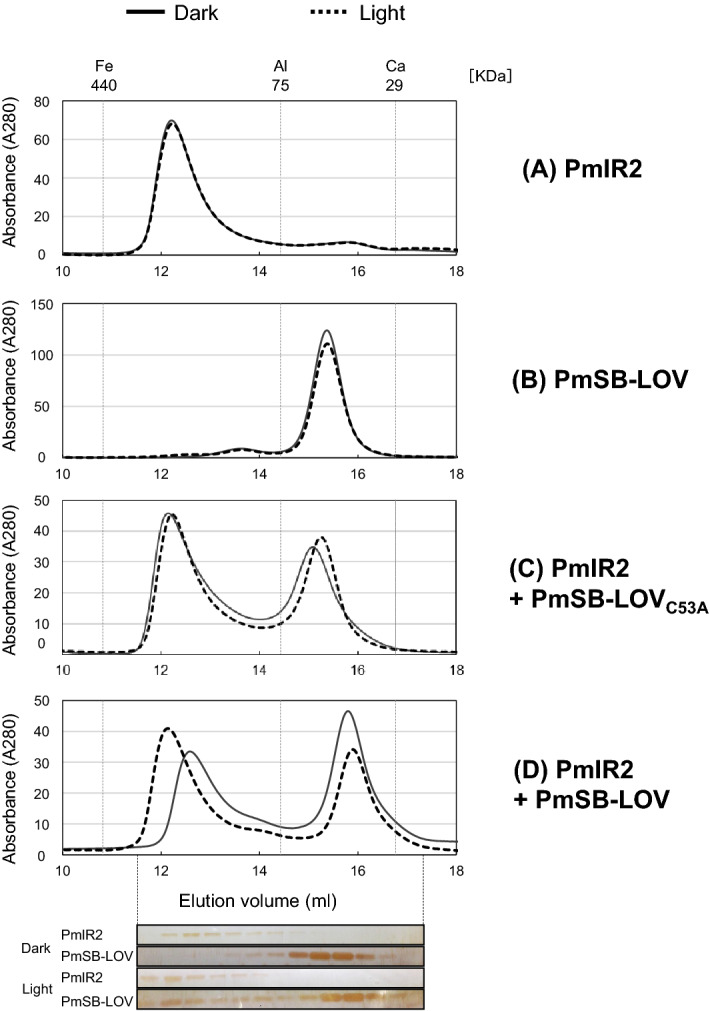
Protein–protein interaction between PmlR2 and PmSB-LOV by gel-filtration chromatography. The recombinant PmlR2 (**A**), PmSB-LOV (**B**), PmSB-LOV_C53A_ and PmlR2 (**C**), and PmSB-LOV and PmlR2 (**D**) incubated under dark (solid line) and light (dashed line) conditions were applied to a Superdex 200 HR 10/30 column on an ÄKTA FPLC system. PmlR2 (200 µL; 0.43 mg/mL), PmSB-LOV (200 µL; 0.47 mg/mL), PmSB-LOV_C53A_ (200 µL; 0.21 mg/mL) recombinant proteins were used. The column was equilibrated and developed with 1 × PBS at a flow rate of 0.25 mL/min. Molecular size standards (Fe: Ferritin, Al: Conalbumin, and Ca: Carbonic anhydrase indicating 440, 75, and 29 kDa, respectively) were applied. White light was applied at approximately 50 µmol s^−1^ m^−2^ for 5 min. SDS-PAGE and silver staining for panel D is shown at the bottom. Two independent experiments in technical replicates were performed, and representative data were shown. The original and unprocessed versions of the full-length stained gels were included in Fig. S13.

To confirm the light-induced interaction between PmSB-LOV and PmlR2 observed in the gel-shift assay, the gel-filtration chromatography analysis was performed. Single proteins or protein mixtures incubated under dark or light conditions were separated using gel-filtration chromatography. When each protein was separately loaded onto the column, PmlR2 and PmSB-LOV eluted at approximately 226 kDa and 51.8 kDa, regardless of their illumination state (Fig. 6A and B), suggesting that PmlR2 and PmSB-LOV exist in a hexamer and trimer, respectively, in solution.

When PmlR2 and PmSB-LOV were co-incubated in the dark, they were eluted as two major peaks (Fig. 6D). Each elution volume was almost identical to those of PmlR2 and PmSB-LOV. SDS-PAGE and silver staining confirmed that the former and latter peaks contained PmlR2 and PmSB-LOV, respectively. Thus, incubation in dark did not affect the interaction between PmSB-LOV and PmlR2. In contrast, illuminated mixtures of PmlR2 and PmSB-LOV generated a new peak (Fig. 6D) that eluted at a higher volume. The new peak contained PmlR2 and PmSB-LOV, whereas the latter two peaks were eluted in almost same fraction as PmlR2 and PmSB-LOV, respectively. Therefore, PmlR2 and PmSB-LOV interacted in response to illumination. We examined PmSB-LOV_C53A_ using the same method. As shown in Fig. 6C, PmSB-LOV_C53A_ did not associate with PmlR2 under dark or light conditions. Our results indicate that PmSB-LOV directly associates with PmlR2 in response to illumination, and Cys^53^ may be involved in this light-dependent association.

### N-terminal domain of PmlR2 interacts with PmSB-LOV

To determine the interaction domain between PmlR2 and PmSB-LOV, two truncated proteins, (i) PmlR2-N protein containing only the HTH DNA-binding domain and (ii) PmlR2-C protein containing only the C-terminal domain, were subjected to gel-filtration chromatography analysis. The calculated molecular weights of the PmlR2-N and PmlR2-C recombinant proteins with the GPLGS sequence were 12.8 kDa and 25.9 kDa, respectively. When each protein was separately loaded onto the column, PmlR2-N and PmlR2-C eluted at approximately 22 kDa and > 440 kDa, respectively, regardless of the illumination state (Figs. S6 and S7), suggesting that PmlR2-N and PmlR2-C likely exist as dimers and oligomers, respectively. When PmlR2-N and PmSB-LOV were co-incubated in the dark, they eluted as two major peaks. Each elution volume was almost identical to those of PmlR2-N and PmSB-LOV (Fig. S6). In contrast, the illuminated mixtures of PmlR2-N and PmSB-LOV generated a new peak (Fig. S6), which eluted at a higher volume and contained both PmlR2 and PmSB-LOV. Therefore, PmlR2-N interacted with PmSB-LOV in response to illumination. We examined the interaction between PmlR2-C and PmSB-LOV using the same method. As shown in Fig. S7, PmlR2-C is not associated with PmSB-LOV under dark or light conditions. Our results indicate that the PmlR2-N domain directly interacts with PmSB-LOV in response to illumination and also suggests that the PmlR2-N domain prevents oligomerization of its C- terminal domain.

### Light-dependent inhibition of the PmlR2 DNA-binding activity by PmSB-LOV

Our previous genetic study on *P. putida* KT2440 suggested that PplR serves as a negative regulator, and its activity is controlled by PpSB-LOV. Therefore, we studied the function of PmSB-LOV by examining whether PmlR2 DNA-binding activity is inhibited in the presence of PmSB-LOV using surface plasmon resonance spectroscopy (BIAcore).

**Figure 7:**
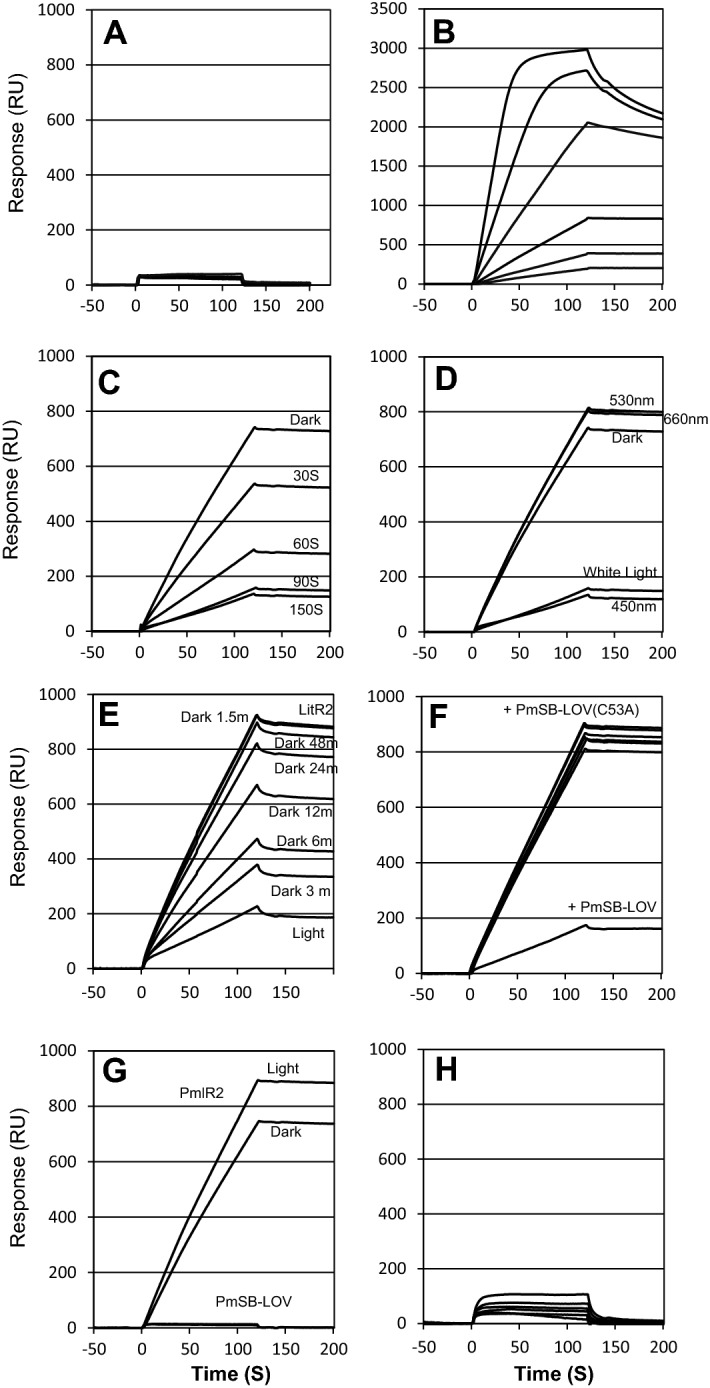
BIAcore analysis. Immobilized DNA on the surface of the sensor chip included P*468* as a negative control (**A**), PmlR2-binding Site 2 (**B**)–(**G**), and the mutated PmlR2-binding Site 2 (**H**). Injected proteins were 8.6 to 275.2 nM PmlR2 (**A**, **B** and **H**), 34.4 nM PmlR2 and 292.3 nM PmSB-LOV (**C**–**E**, and** G**), 34.4 nM PmlR2 and 292.3 nM PmSB-LOV_C53A_ (**F**). Samples were irradiated with blue light (λ_max_ = 450 nm), green light (λ_max_ = 530 nm), and red light (λ_max_ = 660 nm) at 100 µmol m^−2^ s^−1^ for the indicated times. Two independent experiments in technical replicates were performed, and representative data were shown.

The promoter region preceding *PP_0468* was used as a negative control since PmlR2 did not bind to this region in gel-shift assays (data not shown). A biotin-conjugated oligonucleotide containing PmlR2-binding Site 2 of P*6650* (− 9 to + 41, Fig. 2A) was fixed to the sensor chip SA. No PmlR2-binding response was observed when P*468* was immobilized on the sensor chip (Fig. 7A). In contrast, a dose-dependent increase in response units was observed following the infusion of increasing amounts of PmlR2 over Site 2 bound to the immobilized chip (Fig. 7B), indicating that PmlR2 bound to Site 2.

We examined the effect of PmSB-LOV on the DNA-binding activity of PmlR2 (Fig. 7C). The PmlR2 DNA-binding activity was inhibited in the presence of illuminated PmSB-LOV. This inhibition was caused by blue-light illumination for 90 s; in contrast, dark incubation or illumination with 365 nm, green, or red light had no effect (Fig. 7D). We also investigated the dark state recovery of the PmSB-LOV after illumination with blue light. A large increase in response units was observed, representing PmlR2-binding during a long dark incubation period (Fig. 7E). Thus, dark incubation caused a loss of inhibitory function due to dark state recovery of PmSB-LOV.

We also examined the function of PmSB-LOV_C53A_ (Fig. 7F), which did not inhibit the DNA-binding activity of PmlR2. We confirmed that illumination did not affect the DNA-binding affinity of PmlR2 and that PmSB-LOV has no DNA-binding activity (Fig. 7G). We also confirmed specific binding of PmlR2 to Site 2 (TGTACA-N_12_-TGTACA). PmlR2 did not bind dsDNA containing a mutated Site 2 (AAGCTT-N_12_-AAGCTT), in which both consensus TGTACA sequences were replaced with AAGCTT (Fig. 7H). Our data suggest that the DNA-binding activity of PmlR2 is inhibited by blue-light activated PmSB-LOV, and Cys^53^ is required for its inhibitory function. Moreover, dark incubation of PmSB-LOV eliminates the inhibitory function due to dark state recovery.

### PmSB-LOV serves as an anti-repressor against PmlR2

We suggest that PmSB-LOV senses blue light and associates with PmlR2 to inhibit its DNA-binding activity. To clarify whether PmSB-LOV directly inhibits PmlR2 function in vitro, we performed an in vitro transcriptional runoff assay using components required for mRNA synthesis and regulation. We used RNA polymerase holoenzyme containing an essential sigma factor σ^70^, a commercially available protein derived from *E. coli*, because the -10 and − 35 sequence of P*6650* is similar to that of the *E. coli* σ^70^-dependent promoter. *Pseudomonas* σ^70^ is insoluble and, thus, could not be used in this study.

**Figure 8:**
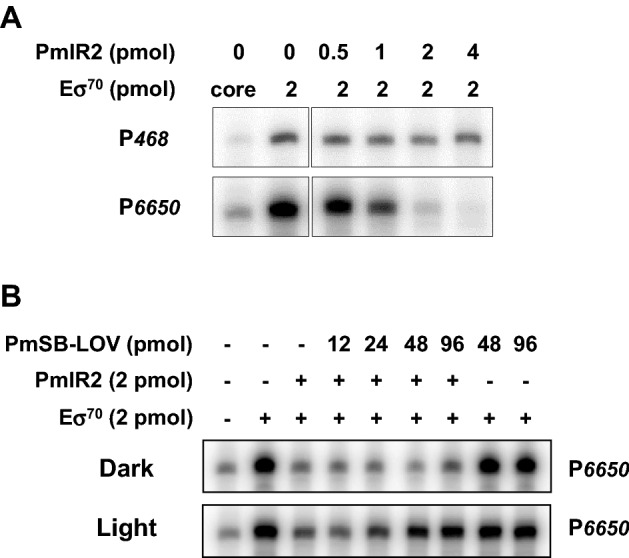
In vitro transcriptional runoff assay. (**A**) The assay for the repressor activity of PmlR2. Templates contained P*468* for the control and P*6650* including the PmlR2-binding site. (**B**) The anti-repressor activity of PmSB-LOV. P*6650* was used as a template. The RNA polymerase holoenzyme containing σ^70^ (E σ^70^) or its coreenzyme, PmlR2, and PmSB-LOV were added to the reaction mixture with 0.5 pmol template in the indicated amount, and the proteins were marked as presence (+) or absence (−). The generated transcripts for P*468* and P*6650* were analyzed by 6 M urea non-denaturing PAGE, followed by autoradiography. Two independent experiments in technical replicates were performed, and representative data were shown. The original and unprocessed versions of the full-length gels were included in Fig. S14 and S15.

The RNA polymerase holoenzyme generated a transcript of the expected length from the template containing P*6650* or P*468* promoters (Figs. 8A, S8), whereas the core enzyme of the *E. coli* RNA polymerase produced small amount of transcript. To examine whether PmlR2 serves as a repressor, we added PmlR2 to the transcription reaction. PmlR2 dose-dependently inhibited the mRNA synthesis from P*6650*. Inhibition was not observed when P*468* was used as a template; thus, PmlR2 specifically inhibited the transcription from P*6650*.

We then assessed whether PmSB-LOV has a negative regulatory effect on PmlR2 activity in response to light. Illuminated or non-illuminated PmSB-LOV were added to the transcription reaction in the presence of PmlR2. The addition of illuminated PmSB-LOV restored transcription in a dose-dependent manner despite the presence of PmlR2 (Figs. 8B, S8). However, the presence of non-illuminated PmSB-LOV did not affect the transcriptional efficiency. No inhibition was observed in the presence of PmSB-LOV. Our results demonstrated that PmlR2 serves as a repressor and that PmSB-LOV serves as a light-dependent anti-repressor of PmlR2 activity.

## Discussion

The biochemical properties of the bacterial “short” LOV proteins have been well studied, but their effector proteins have not been identified. Our study revealed that the MerR family transcriptional regulator PmlR2 is an effector protein of PmSB-LOV in *P. mendocina* NBRC14162. This is the first report on the identification of a protein partner for a “short” bacterial LOV protein. Furthermore, in the bacterial photo-response system, the molecular mechanism underlying the interaction between the MerR family regulator and the LOV is unknown. Therefore, the regulatory system identified in this study is a novel photo-regulatory mechanism. The conservation of PmlR2 and PmSB-LOV homologs in *Pseudomonas* spp. and closely related bacteria indicates that light-sensing and responses are required for survival in nature.

**Figure 9:**
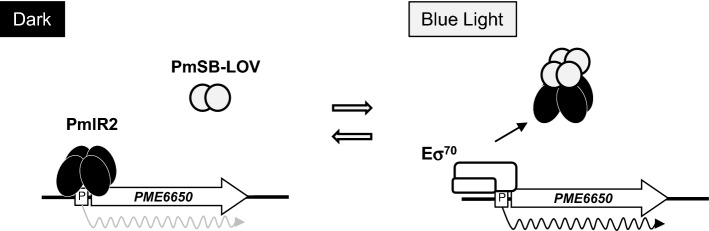
Model for light-inducible transcriptional control by PmlR2 and PmSB-LOV. PmlR2 binds to the promoter region of the light-inducible P*6650* and prevents transcriptional initiation by RNA polymerase holoenzyme containing σ^70^. Upon illumination, PmSB-LOV becomes active and forms a covalent thiol-adduct with FMN. Activated PmSB-LOV associates with PmlR2, abolishing its DNA-binding activity and allowing RNA polymerase to initiate transcription from P*6650*. Dark incubation causes the covalent FMN-C4a-cysteinyl-thiol adduct to break, leading to dissociation of PmlR2 and PmSB-LOV and inhibition of P*6650* by the binding of PmlR2.

Figure 9 illustrates the regulatory model based on the results of this study. Under dark conditions, PmlR2 associates with binding sites in the promoter region of *PME6650*, preventing access by RNA polymerase. Upon illumination with blue light, FMN-bound PmSB-LOV is converted to its activated form, which interacts directly with PmlR2. This protein–protein interaction abolishes the DNA-binding activity of PmlR2, thus permitting transcription from the light-inducible promoters by RNA polymerase. When the environment returns to dark conditions, PmSB-LOV becomes inactivated due to dark-state recovery, and free PmlR2 binds to the promoter region, inducing the OFF state.

The in vitro transcriptional runoff assay clearly demonstrated that the base function of PmlR2 is a repressor, which is fundamental to preventing the expression of light-inducible genes when they are not required, similar to the function of many LitR/CarH family members. PmlR2 bound to a consensus sequence similar to that of PplR1 (Fig. 2C) and the N-terminal HTH DNA-binding domain of PmlR2 was highly similar (78%) to that of PplR1. Consensus was also found 95 bp upstream of the translational initiation site (5′-TGTACA-N_12_-GGTATA-3') for the light-inducible gene, *folM*. Thus, PmlR2 may bind to these sequences to control their transcription. Comprehensive analysis such as chip-seq will permit a more thorough evaluation of PmlR2 binding sites in future studies.

PmlR2 contains an internal B_12_ binding domain and C-terminal domain of unknown function (Fig. S1A). Many previous studies have shown that the B_12_-dependent photosensor LitR of *T. thermophilus* has two C-terminal B_12_-binding domains (B_12_-binding_2 and B_12_-binding domain) that mediate AdoB_12_-binding^1,27^. The latter domain contains a motif essential for the association with B_12_. The internal domain of PmlR2 (residues Trp^113^ to Leu^161^) is similar to the B_12_ binding_2 domain with an *E*-value of 1.1e-05, while the C-terminal domain of PmlR2 has low similarity (19.3%) to the B_12_-binding domain of *Thermus* LitR. The low similarity with the B_12_-binding domain suggests that PmlR2 does not serve as an AdoB_12_-binding domain. Consistent with this, our previous study on *P. putida* KT2440 showed that B_12_ is not required for light-inducible transcription via PplR proteins, the PmlR2 homolog^9^. We assume that the C-terminus serves as an oligomerization domain based on the results of gel-filtration analysis (Fig. S7).

The PmSB-LOV was predominantly associated with FMN (Fig. S4) and exhibited typical LOV domain-type properties such as light-induced spectral changes and dark state recovery (Fig. 3A). The adduct state lifetime of PmSB-LOV was similar to that of PpSB1-LOV in *P. putida* KT2440 (109 ± 18 min for PpSB1-LOV, 0.5 ± 0.1 min for PpSB2-LOV). The dark state recovery of PmSB-LOV was also observed in the BIAcore analysis (Fig. 7E).

The conformation of the well-studied “short” LOV protein is largely affected by illumination: RsLOV of *Rhodobacter sphaeroides* shows light-induced subunit dissociation^28^. In contrast, PmSB-LOV and PpSB1-LOV^16–18^ constitutively formed dimers in solution, regardless of the light conditions (Fig. 6). The light-state crystal structure of PpSB1-LOV suggests that the N-terminal N-cap and C-terminal Jα-helix located at the subunit interface stabilize the parallel homodimer via coiled-coil interactions. The N-cap and Jα-helix are strictly conserved among “short” LOV proteins, including PmSB-LOV (Fig. S1B), suggesting that a light-induced conformational change, but not subunit dissociation or association, is necessary for activity switching in PmSB-LOV. The three-dimensional structures of the protein in dark and light will reveal the molecular mechanism underlying the functional changes in response to illumination.

The conserved Cys^53^ residue was essential for light-inducible activation of PmSB-LOV, as it was required for the light-induced absorption spectrum change (Fig. 3A), the interaction with PmlR2 (Fig. 6), and PmlR2 inhibitory activity (Figs. 7, 8). In contrast, the Cys^53^ residue was not involved in the association with FMN (Figs. 3A and S4) or dimerization (Fig. 6). These results indicate that the formation of a covalent bond between Cys^53^ and position 4a of the flavin chromophore is the foundation of the light-induced functional change in PmSB-LOV, generating the active form of PmSB-LOV upon light excitation. Thus, the Cys^53^ residue is crucial to light-sensing by PmSB-LOV.

Our analysis revealed that the N-terminal HTH domain of PmlR2 interacts with PmSB-LOV (Fig. S6). The short N-terminal extension and Jα-helix of the LOV domain of the EL222 protein in the marine bacterium *Erythrobacter litoralis* interact with an HTH domain in its C-terminus^29^. This suggests that the Jα-helix of PmSB-LOV (Fig. S1B) interacts with the HTH domain of PmlR2 (Fig. S1A). Further analysis is required to characterize the molecular mechanism underlying the interaction.

Our results provide the first evidence of interaction between a “short” LOV protein and partner protein, since gel-shift assay (Fig. 5) and gel-filtration chromatography (Fig. 6) demonstrated the light-induced protein–protein interaction between PmlR2 and PmSB-LOV. Furthermore, we report that an LOV functions as an anti-repressor, since BIAcore analysis demonstrated that illuminated PmSB-LOV inhibits the DNA-binding activity of PmlR2 (Fig. 7), and the in vitro transcriptional runoff assay demonstrated that blue-light-activated PmSB-LOV serves as an anti-repressor of PmlR2 (Fig. 8). We also observed dark recovery with BIAcore analysis and found that the time required for dark recovery was similar to the absorbance spectra after 60 min. Our study revealed that PmSB-LOV directly associates with PmlR2 in a light-inducible manner; however, the intramolecular mechanism and structure of PmSB-LOV are unknown. Elucidation of the three-dimensional structures of the dark and light states may shed light on the precise light-response mechanism.

The gel-shift assay and BIAcore analysis showed different results in the complex formation of PmlR2, PmSB-LOV, and probe DNA under light conditions. The gel-shift assay showed the formation of a stable PmlR2/PmSB-LOV complex bound to P*6655* (right panel of Fig. 5), while BIAcore analysis showed inhibition of PmlR2 DNA-binding activity by PmSB-LOV (Fig. 7C). In both experiments, the PmlR2/PmSB-LOV complex was formed under light conditions in advance, and followed by analysis of the interaction with the probe. In the gel-shift assay, gels were continuously illuminated with light from an external source for approximately 120 min of electrophoresis. In contrast, in BIAcore analysis, the time required for the protein complex to come into contact with the probe was 1–2 min. Therefore, the inconsistency of the results between the two experiments may be due to the different experimental conditions. We favor the result of the BIAcore analysis as it requires less analysis time.

This study also provides insights into the role of MerR family regulator. Two types of MerR family proteins involved in the light-response system of bacteria have been identified: (i) the light-sensitive AdoB_12_-based LitR/CarH family and (ii) *E. coli* BluR controlled by the blue light receptor BluF^30^. Our study reveals a new type, (iii) PmlR2 regulated by a “short” LOV protein. The wide distribution of LitR/CarH family homologs leads us to assume the diversity of an unidentified photo-response mechanisms in bacteria.

## Materials and methods

### Bacterial strains, plasmids, and culture media

*Pseudomonas mendocina* NBRC 14162^22^ was obtained from the Biological Resource Center, NITE (NBRC), Kazusa, Japan. *Escherichia coli* (*E. coli*) strains HST08 and Rosetta2(DE3)pLysS (Takara Bio Inc., Shiga, Japan) were used as hosts for DNA manipulation and protein expression, respectively. pUC118 (Takara Bio) was used for general DNA manipulation. pMD19 (Takara Bio) was used for TA-cloning of PCR-generated DNA fragments. pGEX-6P-2 (GE Healthcare UK Ltd, Buckinghamshire, England) was used to overexpress PmlR2 and PmSB-LOV in *E. coli.* Enzymes for DNA manipulation were purchased from Takara Bio. Culture conditions and genetic manipulations in *E. coli* are described by Maniatis et al.^31^ *E. coli* and *P. mendocina* were grown at 28 °C or 30 °C in Luria–Bertani (LB) medium^31^; 1.5% agar (Kokusan, Tokyo, Japan) was added to the solid media. Antibiotics were added to the following final concentrations: ampicillin and kanamycin at 50 μg/mL; chloramphenicol at 15 μg/mL.

### Light irradiation experiments

An illuminating incubator (BR-180LF; Taitech, Saitama, Japan) equipped with white, blue, and red fluorescent lamps (20 W; Toshiba, Tokyo, Japan) was used for shaking liquid culture. The purified recombinant proteins were irradiated with LED lamps, blue light (λ_max_ = 450 nm), green light (λ_max_ = 530 nm), and red light (λ_max_ = 660 nm) (Optocode, Tokyo, Japan). Radiance was measured with a Model LI-250 Light Meter (LI-COR Inc., Lincoln, NE, USA).

### Total RNA preparation and quantitative RT-PCR

Total RNA was purified from *P. mendocina* with an RNeasy Protect Bacteria kit (Qiagen GmbH, Hilden, Germany) according to the manufacturer’s instructions. *P. mendocina* was cultured at 28 °C in liquid LB under blue light (30.5 µmol s^−1^ m^−2^) and dark conditions. cDNA was synthesized with SuperScript VILO cDNA Synthesis Kit (Thermo Fisher Scientific) as described by the manufacturer. The amount of the synthesized cDNA was quantified with PowerUp SYBR Green Master Mix (Thermo Fisher Scientific) and an Applied Biosystems 7500 Real-Time PCR System (Thermo Fisher Scientific) according to the manufacturer’s instructions and our previous study^8^. The oligonucleotide primers were summarized in Table S2. Relative gene expression was quantified using the signals of *dnaA* (an essential chromosomal replication initiator gene) as an internal reference and the relative quantitative 2^−ΔΔCt^ method^32^. All reactions were performed in triplicate.

### RNA-seq analysis

For sequencing library preparation, 1 µg of total RNA was prepared using the following methods. Ribosomal RNA was removed using the RiboZero bacteria kit (Illumina, San Diego, CA, USA), according to the manufacturer’s protocol. Sequencing libraries were prepared by NEBNext mRNA Library Prep Kit from Illumina (New England Biolabs, Ipswich, MA, USA) with the following modifications. A random hexamer primer was used for reverse transcription. After second-strand synthesis, the double-stranded cDNA was fragmented to an average length of 300 bp using a Covaris S2 sonication system (Covaris, Woburn, CA, USA). One hundred cycles of paired-end sequencing were carried out using HiSeq2500 system according to the manufacturer’s specifications (Illumina). After sequencing reactions were complete, the Illumina analysis pipeline (CASAVA 1.8.0) was used to process the raw sequencing data. The RNA-Seq reads were trimmed using the CLC Genomics Workbench ver. 9.0.1 (Qiagen, Venlo, Netherlands) with the following parameters; Quality score: 0.05; Removing terminal 15 nucleotides from 5’ end and 2 nucleotides from 3’ end; Trimming read through adaptor: yes; Removing truncated reads less than 30 nucleotides in length. Trimmed reads were mapped to all genes in *P. mendocina* NBRC 14162 (accession number: NZ_BBQC00000000.1) using the CLC Genomics Workbench ver. 9.0.1. with the following parameters; Length fraction: 0.7; Similarity fraction: 0.9; Maximum number of hits for a read: 1. The expression level of each gene was calculated by counting the mapped reads for each gene and were normalized by calculating RPKM values. Original sequence reads were deposited to DRA/SRA database under the accession number (DRA014932). The biological replicate in the RNA-seq experiment was *n* = 1, because the purpose of RNA-seq in this study was for preliminary screening of light-inducible genes.

### Determination of transcriptional start sites

Transcriptional start sites were determined by 5′-RACE (rapid amplification of cDNA ends) and CAGE experiments using the total RNA purified from light-irradiated cells. For 5′-RACE, sequencing of the 5′-ends of the *PME6650* and *PP0468* transcripts was carried out with the 5′-Full RACE Core Set (Takara Bio) according to the manufacturer’s instructions. The used oligonucleotides, ORF4-RT, ORF4-A1, ORF4-A2, ORF4-S1, and ORF4-S2 for *PME6650*, and PP468-RT, PP468-A1, PP468-A2, PP468-S1, and PP468-S2 for *PP0468* were shown in Table S3. The amplified DNA fragments were cloned into the T-vector, pMD19 and the cloned inserts were sequenced by Eurofins Genomics. For the CAGE experiment, CAGE library preparation, sequencing, mapping, and gene expression and motif discovery analysis were performed using DNAFORM (Yokohama, Kanagawa, Japan). In brief, RNA quality was assessed using a Bioanalyzer (Agilent) to ensure that RNA integrity number (RIN) is over 7.0, and the A260/280 and 260/230 ratios are over 1.7. First strand cDNAs were transcribed to the 5’ end of capped RNAs, attached to CAGE "bar code" tags and the sequenced CAGE tags were mapped to the *P. mendocina* genomes using BWA software (v0.5.9) after discarding ribosomal RNAs. For tag clustering, CAGE tag count data were clustered using CAGEr^33^ and the Paraclu algorithm^34^ with default parameters. Clusters with count per million (CPM) < 0.2 were discarded.

### Overexpression and purification of recombinant PmlR2, PmSB-LOV, and PmSB-LOV_C53A_

To construct the protein expression vectors for PmlR2, PmlR2-N (1-113 aa), PmlR2-C (95 aa to the C terminus), and PmSB-LOV, each gene was amplified by PCR with primers R2ex-F/R2ex-R (PmlR2), R2ex-F/R2ex-RN (PmlR2-N), R2ex-FC /R2ex-R (PmlR2-C), and LOVex-F/LOVex-R (PmSB-LOV) (Table S3). The expression vector for PmSB-LOV_C53A_, in which Cysteine 53 (Cys^53^) was replaced with alanine, was generated using two-stage PCR. The upstream and downstream regions of the mutation were amplified using the primers LOVex-F/C53A-MR and C53A-MF/LOVex-R, respectively. The first-round PCR amplicons served as a template for the second-round PCR with the primers LOVex-F/LOVex-R. The 3' ends of the upstream first-round PCR products were complementary to the downstream region, thus generating a fusion construct in the second-round PCR. The amplified DNA fragments were digested with *Bam*HI and *Eco*RI, and then cloned into the same site of pGEX-6P-2 (GE Healthcare) to generate pGEX-PmlR2 (PmlR2), pGEX-PmlR2-N(PmlR2-N), pGEX-PmlR2-C(PmlR2-C), pGEX-LOV (PmSB-LOV), and pGEX-C53A (PmSB-LOV_C53A_). The insertions were verified by sequencing using an ABI3100 Genetic Analyzer. The recombinant gene encodes a protein with N-terminal GST under the control of an isopropyl β-D-thiogalactoside (IPTG)-inducible promoter. *E. coli* Rosetta2(DE3)pLysS cells harboring the expression vectors were cultured in LB liquid medium at 28 °C and IPTG was added when the culture reached 0.3 of an OD_600_. After 3 h induction, cells were harvested by centrifugation and suspended in 1 × PBS buffer (containing 140 mM NaCl, 2.7 mM KCl, 10 mM Na_2_PO_4_, and 1.8 mM KH_2_PO_4_). Cells were disrupted with a sonicator (Astrason XL2020; Misonix, NY, USA). The cell debris was removed by centrifugation, and the supernatant was transferred to a 5 mL GSTrap HP column (GE Healthcare) on an ÄKTA explorer 10S (GE Healthcare). The N-terminal GST tag was removed by PreScission protease (GE Healthcare) at 4 °C for 16 h on a GST affinity column. Proteins were eluted and dialyzed against 1 × PBS. The eluted recombinant proteins have an additional N-terminal GPLGS sequence prior to the translational initiation codon. After homogeneity was verified by SDS-PAGE with Coomassie or silver staining, the purified proteins were divided into small volumes, and stored at 4 °C or − 20 °C until further use. Absorption spectra were recorded by a UV spectrometry on a U-2800A (Hitachi High-Tech Science Corp., Tokyo, Japan), Nano Drop 2000 (Thermo Fisher Scientific), or Cary 8454 UV–Vis Diode Array System (Agilent Technologies, CA, USA). Protein concentrations were determined by the Bradford method as described in the instructions accompanying the Bio-Rad protein assay kit (Bio-Rad Laboratories, CA, USA) with BSA (Thermo Fisher Scientific) as a standard.

### HPLC analysis of chromophore

The flavin derivative chromophore of PmSB-LOV was analyzed as previously described^35^. The chromophore of PmSB-LOV (4.7 µg) and PmSB-LOV_C53A_ (12 µg) were released by heat denaturation at 95 °C for 10 min. The samples were then centrifuged at 14,000 rpm for 20 min. After filtration (0.22 µm filter), 10 µL samples were injected into a HPLC equipped with a CAPCELL PAK C18 column (SISEIDO, Tokyo, Japan). Ammonium acetate (50 mM, pH 6.0) containing 70% acetonitrile was used as the solvent. Commercially available flavin mononucleotide (FMN) and flavin-adenine dinucleotide (FAD) (Wako, Tokyo, Japan) were used as reference compounds.

### Spectral analysis

PmSB-LOV protein absorbance spectra were recorded with Cary 8454 UV–Vis Diode Array System. Absorbance spectra (300–600 nm) of the illuminated PmSB-LOV proteins were recorded after the sample was irradiated with a blue light-emitting LED lamp at approximately 100 µmol s^−1^ m^−2^ for 10 s. The dark state recovery was measured every 20 s for 1 h at 480 nm with 1 × PBS buffer used as a reference. All measurements were performed at 25 °C in 10-mm light-path quartz cuvettes.

### Gel-shift assay

Gel-shift assay were performed as previously described^9^. The probes for P*6650*, P*rpoD* and P*folM* were amplified by PCR with primer pair, ORF4-FPF/ORF4-FPR, rpoDF/rpoDR and ORF12F/ORF12R, respectively (Table S3). The DNA fragments were phosphorylated with T4 polynucleotide kinase and [γ-^32^P] ATP. The reaction mixture containing a total of 0.5–5.0 ng of ^32^P-labeled probes (10,000–20,000 cpm), 0–160 pmol of recombinant PmlR2, 0–12 pmol of PmSB-LOV in 50 µL of binding buffer [containing 10 mM Tris–HCl (pH 7.0), 50 mM KCl, 1 mM EDTA, 1 mM dithiothreitol, 10% (vol/vol) glycerol, 1 µg poly(dI-dC), and 50 µg mL^−1^ of bovine serum albumin] was incubated at 37 °C for 30 min. Poly(dI-dC) was used as competitor for non-specific DNA-binding proteins. For the super-shift assay, PmlR2 and PmSB-LOV were incubated under light conditions (42 µmol m^−2^ s^−1^) for 10 min, and then ^32^P-labeled probes and poly(dI-dC) were added to the reaction mixtures. The mixture was further incubated under light for 30 min. During electrophoresis, native PAGE was continuously illuminated with 42 µmol m^−2^ s^−1^ white light at room temperature. Radioactivity in the dried gels was detected by exposure to a Fuji imaging plate (Fuji Film, Tokyo, Japan), and images were scanned with a Typhoon 9410 or Typhoon FLA 9500 image analyzer (GE Healthcare).

### DNase I footprint analysis

To determine the PmlR2 DNA-binding site, DNase I footprint analysis was performed as described previously^9^. To label the sense or antisense strands of DNA, one primer was phosphorylated with T4 polynucleotide kinase and [γ-^32^P] ATP. ^32^P-labeled DNAs fragments were prepared by PCR amplification with primers ORF4-FPF*/ORF4-FPR (Table S3) for the sense strand and ORF4-FPF/ORF4-FPR* for the antisense strand. Asterisks indicate primers labeled with [γ-^32^P] ATP. The reaction mixture (50 μL) was composed of 10 kcpm ^32^P-labeled DNA probe; 10–80 pmol PmlR2; 25 mM HEPES–KOH, pH 7.9; 0.5 mM EDTA-NaOH, pH 8.0; 50 mM KCl; and 10% glycerol. To bind PmlR2 to the probe, incubation was performed at 25 °C for 30 min. DNase I was added at a final concentration of 20 μg/ml, and the mixture was further incubated for 1 min. To terminate the reaction, 100 μL of stop solution (containing 100 mM Tris–HCl, pH 8.0; 100 mM NaCl; 1% sodium *N*-lauroyl sarcosinate; 10 mM EDTA-NaOH, pH 8.0; and 25 mg/ml salmon sperm DNA) and 300 μL of phenol:chloroform (1:1) were added. The digested probes were concentrated using ethanol precipitation, and then the pellet was washed with 80% ethanol, dissolved in a 6 μL formamide-dye mixture, and run on a 6% polyacrylamide gel. Visualization by autoradiography was performed as described above. For reference, Maxam–Gilbert sequencing ladders (G + A and T + C reactions) were prepared from the ^32^P-labeled probe DNA^36^.

### Bacterial adenylate cyclase two-hybrid (BACTH) system analysis

The protein–protein interaction between PmlR2 and PmSB-LOV was investigated with a BACTH system kit (EUROMEDEX, Souffelweyersheim, France)^37^ according to the manufacturer’s instructions. The genes encoding PmlR2 and PmSB-LOV were PCR-amplified with primers R2T-F/R2T-R (Table S3) for *pmlR2* and LOVT-F/LOVT-R (Table S3) for *pmSB-LOV*. The amplicons were digested with *Xba*I and *Kpn*I, and then cloned into the same site of pUT18C and pKT25. PmlR2 and PmSB-LOV were fused at the C-terminus of the split adenylate cyclase domains (T18 and T25). The nucleotide sequence was confirmed by Eurofins Genomics. The pUT18C and pKT25 derivatives were co-transformed into a *cya*-deficient strain of *E. coli* BTH101, supplied by the manufacturer. β-galactosidase assays were performed as previously described^38^.

### Gel-filtration chromatography

To estimate the native molecular weight of the recombinant proteins, gel-filtration column chromatography was performed. Samples of PmlR2 (0.43 mg/mL), PmSB-LOV (0.48 mg/mL), and PmSB-LOV_C53A_ (0.21 mg/mL) were applied in 200-µL volumes to Superdex 200 HR 10/30 column on an ÄKTA FPLC system (GE Healthcare) at a flow rate of 0.3 mL/min according to the manufacturer’s instructions. For interaction analysis, PmlR2 and PmSB-LOV were co-incubated under light conditions (approximately 50 μ mol s^−1^ m^−2^) for 5 min before being applied to the column. The column was equilibrated and developed with cold 1 × PBS containing 140 mM NaCl, 2.7 mM KCl, 10 mM Na2PO4 and 1.8 mM KH2PO4 (pH 7.2) at a flow rate of 0.3 mL/min. A gel-filtration calibration kit (GE Healthcare) was used for the size standards (ferritin, aldolase, conalbumin, ovalbumin, RNase A, and blue dextran, corresponding to 440, 158, 75, 43, 13.7, and 2 kDa, respectively).

### BIAcore analysis

To examine the function of PmlR2 and PmSB-LOV proteins, surface plasmon resonance analysis was performed on a BIAcore 1000 (GE Healthcare). DNA containing wild-type PmlR2-binding site 2, mutated PmlR2-binding site 2, and P*468* (Table S4) was immobilized on the streptavidin surface of a Sensor Chip SA (GE Healthcare). HPLC-grade 5′-biotin-conjugated sense and label-free antisense oligonucleotides (both 50 nt) were prepared. HBS-EP (GE Healthcare) was used as the running buffer at 10 μL/min and 28 °C. PmlR2 (8.6–275.2 nM) and PmSB-LOV (292.3 nM) samples (20 µL) were injected and sensorgrams were recorded. An equivalent volume of each protein dilution was applied to the non-immobilized surface to estimate the background bulk refractive index. The surfaces of Sensor Chip SA were regenerated with 80 µL of HBS-EP containing 2 M NaCl at 100 µL/min. PmSB-LOV and PmSB-LOV_C53A_ were exposed to 100 μ mol m^−2^ s^−1^ white, 365 nm, 450 nm, 530 nm, and 660 nm light for 90 s and the association/dissociation rate constants and the dissociation constant (*Kd*) were estimated by fitting the data to a simple 1:1 Langmuir binding model with BIAevaluation software (version 4.1.1; GE Healthcare).

### In vitro transcriptional runoff assay

The in vitro runoff assay was performed as previously described^3^. Templates containing the promoter and operator sequences were PCR-amplified with the primers 4RO-F/4RO-R for P*6650* (198 bp) and CRO-F/CRO-R for P*468* (303 bp) (Table S3). The template (0.5 pmol) was mixed with 2 pmol *E. coli* RNA polymerase holoenzyme or core enzyme (AR Brown, Tokyo, Japan), 100 nmol ribonucleotide (including [α-^32^P] CTP), 0–4 pmol PmlR2, 0–96 pmol PmSB-LOV. Samples were illuminated with 42 µmol m^−2^ s^−1^ white light at 28 °C for 10 min. Transcripts were analyzed by 6 M urea polyacrylamide gel electrophoresis. Marker 10 (pBR322/*Msp*I digest) (Nippon Gene, Tokyo, Japan) was labeled with [γ-^32^P] ATP and used as a molecular marker. Visualization by autoradiography was performed as described above.

### Significance statement

"Short" LOV proteins are blue-light photosensors that are widely distributed in the genome of *Pseudomonas* spp. Whilst the biochemical functions of "short" LOV proteins have been well studied, any associated effector proteins have not yet been identified. We revealed, for the first time, that PmlR2 belonging to the MerR family transcriptional regulator functions as the partner protein of “short” LOV proteins. The conservation of PmlR2 and PmSB-LOV homologs in *Pseudomonas* spp. and closely related bacteria indicates that light sensing and response are required for survival in nature.

## Supplementary Information

Supplementary Information.

## Data Availability

The datasets of RNA-seq generated during the current study are available in the DRA/SRA database repository, Accession No. DRA014932.
