# Supplementary material for: Class II LitR serves as an effector of “short” LOV-type blue-light photoreceptor in Pseudomonas mendocina
Source: Sci Rep. 2022 Dec 16;12:21765. doi: 10.1038/s41598-022-26254-3 (PMC9758184; doi:10.1038/s41598-022-26254-3)
Supplement: Supplementary file 1 — Supplementary Information. [file 41598_2022_26254_MOESM1_ESM.pdf]

Supplementary Table S1. Light-induced genes of *P. mendocina* identified by RNA-seq analysis.

	Gene name	RPKM ratio Light/Dark[a]	Direction	Annotation for product
aspA	*PME_1590*	2.58	+	aspartate ammonia-lyase
	PME_1595	2.30	+	sodium:dicarboxylate symporter
rpmE	*PME_3835*	2.73	-	50S ribosomal protein L31
	PME_6610	5.0	+	putative esterase/lipase
	PME_6615	2.62	-	membrane protein
ufaM	*PME_6620*	14.34	-	furan-containing fatty acid synthase
	PME_6625	6.48	-	DUF1365 domain-containing protein
	PME_6630	9.15	-	FAD-dependent oxidoreductase
	PME_6635	13.69	-	short-chain dehydrogenase
	PME_6640	14.26	-	transcriptional regulator
phrB	*PME_6645*	12.72	-	deoxyribodipyrimidine photo-lyase
pmlR1	*PME_6650*	4.43	-	MerR family transcriptional regulator
	PME_6655	6.46	-	hypothetical protein
	PME_6670	2.30	+	ferrochelatase
	PME_6905	2.06	+	short-chain fatty acid transporter
	PME_7070	12.30	+	hypothetical protein
cryB	*PME_7075*	7.37	+	cryptochrome/photolyase family protein
capB	*PME_8205*	2.23	-	cold-shock protein CapB
	PME_11530	2.18	-	cyclophilin
	PME_12475	2.0	-	oxidoreductase
folX	*PME_15540*	2.77	-	dihydroneopterin triphosphate 2'-epimerase
folE	*PME_15545*	3.19	-	GTP cyclohydrolase I
folM	*PME_15550*	2.19	-	dihydromonapterin reductase
	PME_16450	6.48	-	aromatic amino acid aminotransferase
phhB	*PME_16455*	7.81	-	4a-hydroxytetrahydrobiopterin dehydratase
phhA	*PME_16460*	7.62	-	phenylalanine 4-monooxygenase
	PME_16545	8.25	+	chemotaxis protein
	PME_16570	6.33	-	short-chain dehydrogenase
	PME_16575	13.30	-	lipoprotein
	PME_16580	9.33	-	hypothetical protein
ccoG	*PME_16670*	4.20	-	cytochrome c oxidase accessory protein CcoG
	PME_16680	4.44	+	sulfite reductase
	PME_16685	4.73	+	oxidoreductase
lrgA	*PME_16690*	2.47	+	murein hydrolase transporter LrgA
rpsU	*PME_17090*	2.29	+	30S ribosomal protein S21
	PME_18685	2.01	-	thiosulfate transporter subunit
	PME_21355	2.52	-	TonB-dependent receptor
	PME_21360	2.11	+	hemin-degrading factor
	PME_21940	11.37	+	4-hydroxyphenylpyruvate dioxygenase
	PME_21945	10.21	+	dioxygenase
	PME_21950	7.55	+	2-keto-4-pentenoate hydratase
	PME_21955	6.36	+	maleylacetoacetate isomerase
	PME_21960	3.38	+	sodium-dependent transporter

[a] Intensity of the WT strain cultured under light condition normalized to that of the wild-type strain cultured under the dark condition.

Supplementary Table S2. Oligonucleotide primers used in qRT-PCR analysis.

Name	Sequence (5′-3′)
pmlR1-F(QRT)	ATGACCAGCGAAACCATTGC
pmlR1-R(QRT)	ATCGGCAGGTAGCCCTCATC
phrB-F(QRT)	CTACAGCCAGTTCCGCAAGG
phrB-R(QRT)	CGGGAATGATGTCGCTCTTC
cfaB-F(QRT)	GGGTCTGCACTGGCTCAATC
cfaB-R(QRT)	TCATGGTCGGGTCGAGAAAC
lov-F(QRT)	AGGGCGAAGACAACATCCTG
lov-R(QRT)	GAAAGCGGCAGTCCTGGTAG
pmlR2-F(QRT)	GCTTGCGTCCAGTGCTTTG
pmlR2-R(QRT)	GACGGGAAACCTCACGAATG
folA-F(QRT)	AGATTGGCTGGCCGAAACAC
folA-R(QRT)	CGTTGAATAGCTGGCGGAAG
folE-F(QRT)	AGGCCATGCAGTACCTTTGC
folE-R(QRT)	CGCACAGCGAGTACAGTTCG
PME_16455-F(QRT)	GCCGAAAGTGTCCGATGAAG
PME_16455-R(QRT)	ATATGATCGCCACGCACCTC
PME_16460-F(QRT)	AGAGCAGCGCGTCTATCTGG
PME_16460-R(QRT)	GGCGTAGAGGGTTTCCTTCG
PME_21940-F(QRT)	CAGCTCCAACGACAATGCTG
PME_21940-R(QRT)	CGTAGAAACCGGACCAGACG
PME_21945-F(QRT)	ACTTCTTCAGCCGCGACAAC
PME_21945-R(QRT)	CGACCTGGCTCTTCTTCAGC
rpoD-F(QRT)	CATTGGTGAGGCGAAAGCTC
rpoD-R(QRT)	GGCCACGGTTGGTGTACTTC
dnaA-F(QRT)	TGTTCCTGTATGGCGGTGTG
dnaA-R(QRT)	CTTGGCATTGGGATTCTTCG

Supplementary Table S3. Oligonucleotide primers used in BACTH, 5’-RACE, construction of expression vector, DNase I footprint, Gel-shift assay, and *in vitro* runoff transcription.

**Name**	**Sequence (5′-3′)** [a]	**Site of Restriction Enzyme** [b]	**Purpose**
R2T-F	CTCGAGTCTAGAGATGCCTGAAGCCATGTCCGAAC	*Xba*I	BACTH
R2T-R	CTCGAGGGTACCCGGGTATCGAGATGACCGGCGAG	*Kpn*I	BACTH
LOVT-F	CTCGAGTCTAGAGATGATCAATGCCAAGCTGCTG	*Xba*I	BACTH
LOVT-R	CTCGAGGGTACCCGGCTTTTCAGGCGCGCCAGTTC	*Kpn*I	BACTH
ORF4-RT	ACCGACCAGGCGTATC	-	RACE RT-primer
ORF4-A1	CACAGGCGCGACAGTTTATG	-	RACE A1-primer
ORF4-A2	CAGCAGGCAGGCGCTGATACC	-	RACE A2-primer
ORF4-S1	GCAGCCTGACCGAACACTTC	-	RACE S1-primer
ORF4-S2	GTTTGCCCGGAAGTCGGCATC	-	RACE S2-primer
PP468-RT	AGGCTTGGCCTCACC	-	RACE RT-primer
PP468-A1	GCTTACGACGGACTTTTCAGC	-	RACE A1-primer
PP468-A2	GATGCGAGTTAGCATGTCC	-	RACE A2-primer
PP468-S1	CTACCCTGAAGGTCGCGGTTG	-	RACE S1-primer
PP468-S2	CTGAAGGACGAAGGTTACATC	-	RACE S2-primer
R2ex-F	GGATCCATGCCTGAAGCCATGTCCGAAC	*Bam*HI	Protein Expression
R2ex-R	GAATTCTCAGGTATCGAGATGACCGGC	*Eco*RI	Protein Expression
R2ex-RN	GAATTCTCACCAGTCGTCACTGCTGAC	*Eco*RI	Protein Expression
R2ex-FC	GGATCCGCCAGCAGTCGCAGTGCCGCC	*Bam*HI	Protein Expression
LOVex-F	GGATCCATGATCAATGCCAAGCTGCTG	*Bam*HI	Protein Expression
LOVex-R	GAATTCTCAGCTTTTCAGGCGCGCCAG	*Eco*RI	Protein Expression
C53A-MR	GCAGAAAGCGGGCGTCCTGGTAG	-	Protein Expression
C53A-MF	CTACCAGGACGCCCGCTTTCTGC	-	Protein Expression
ORF4-FPF	TACCGCGCTGCGCAAAGCAATAC	-	DNase I footprint, Gel-shift assay
ORF4-FPR	TGATACCCAGTTTGGCTTTG	-	DNase I footprint, Gel-shift assay
ORF12F	ATGCAATGCCTGAGCGGCGGAC	-	Gel-shift assay
ORF12R	ACTCAGAACCTGTTCAAAGG	-	Gel-shift assay
rpoDF	TGGAACAACTGCTCAGG	-	Gel-shift assay
rpoDR	CCACCTGTTCCGGATC	-	Gel-shift assay
4RO-F	AACGGCGACCGCTTTCG	-	*In vitro* transcription
4RO-R	TTCAGCACCCAGCAGGCAG	-	*In vitro* transcription
CRO-F	ACGTACCAGGTCTGGTCAAG	-	*In vitro* transcription
CRO-R	TTCAGCCATCTGGGCATTAC	-	*In vitro* transcription

[a] Restriction sites are underlined. [b] Dashes indicate the absence of restriction site.

Supplementary Table S4. Oligonucleotides used in BIAcore analysis.

Name	Sequence (5′-3′) [a]	Promoter region [b]
BIA-1F	GATAGACACAGGTTGTACAAGAACAAGTTTATGTACAAGGTCTTTCGAGG	Wild type Site 2 (+Bio)
BIA-1R	CCTCGAAAGACCTTGTACATAAACTTGTTCTTGTACAACCTGTGTCTATC	Wild type Site 2
BIA-2F	GATAGACACAGGTAAGCTTAGAACAAGTTTAAAGCTTAGGTCTTTCGAGG	Mutated Site 2 (+Bio)
BIA-2R	CCTCGAAAGACCTAAGCTTTAAACTTGTTCTAAGCTTACCTGTGTCTATC	Mutated Site 2
BIA-3F	GTGTCTAGAATGACCGGCTCACCTGAGCCTGGGTTTTTGCCCAAACGGTT	PP_0468 (+Bio)
BIA-4F	AACCGTTTGGGCAAAAACCCAGGCTCAGGTGAGCCGGTCATTCTAGACAC	PP_0468

[a] The consensus sequence required for the PmlR2-binding and the mutated sites are underlined. [b] +Bio indicate the biotin conjugation at 5’-end.

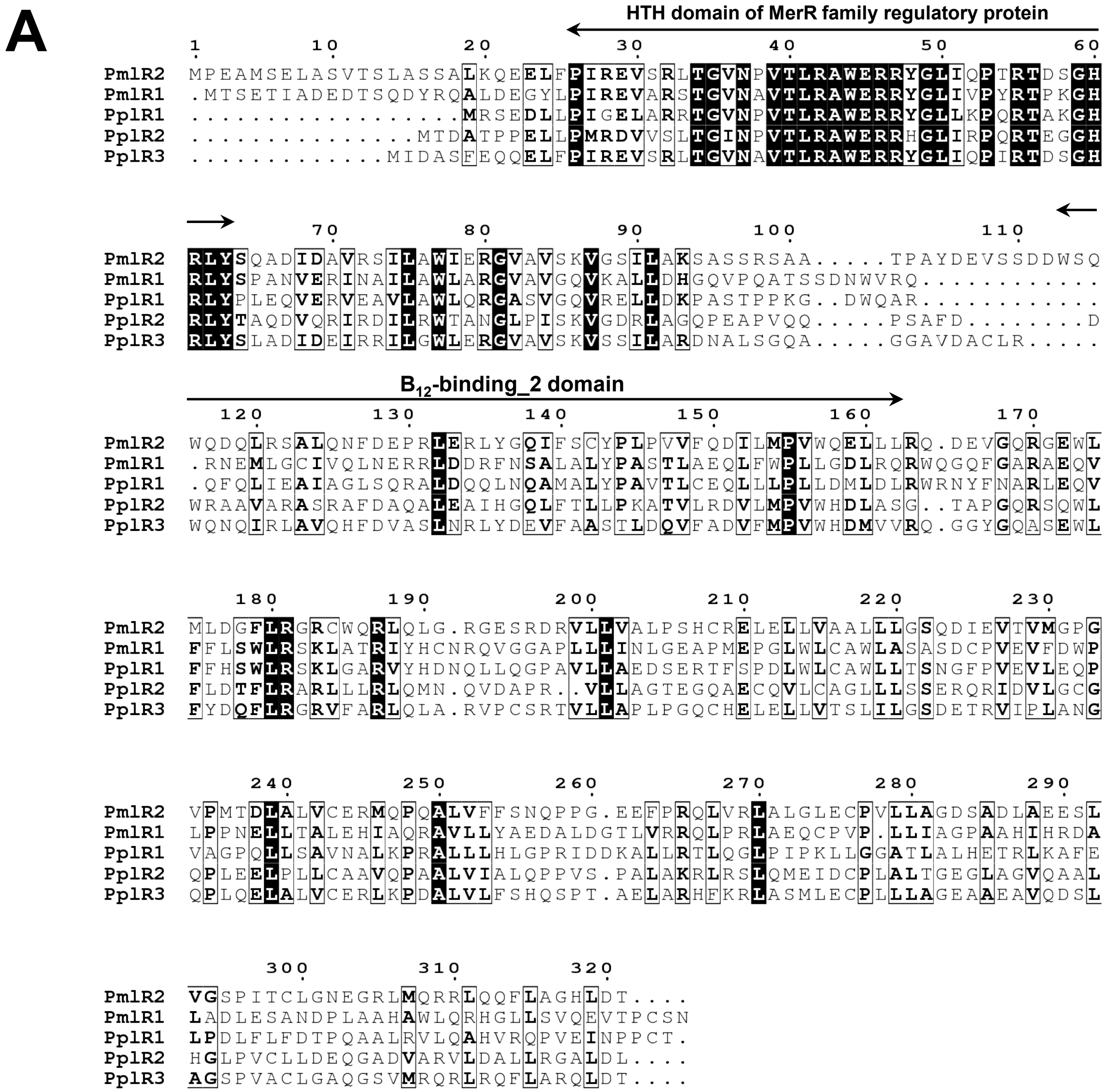

Supplementary Figure S1. Multiple amino acid sequence alignment of PmlR2 (A) and PmSB-LOV (B). The sequences were aligned with CLUSTAL W2 (1) and depicted using ESPrirt 3.0 (2,3). Strictly conserved residues are shown in white letters on a black background, and semi-conserved residues are represented by boxed bold letters. (A) Amino acid sequence alignment of the full-length PmlR2, PmlR1, and PplR1-PplR3 of *P. putida* KT2440. The Helix-Turn-Helix (HTH) and B_{12}-binding_2 domain are indicated by solid and dashed divergent arrows, respectively. (B) The protein structure of PpSB1-LOV based on alignment of “short” LOV proteins from *P. mendocina* NBRC14162 (PmSB-LOV), *P. mendocina* NK-01 (MDS_3586), *P. mendocina* ymp (Pmen_3284), and *P. putida* KT2440 (PpSB1-LOV and PpSB2-LOV). The position of the conserved Cys residue essential for thiol-adduct formation with FMN is indicated with an asterisk. The N-terminal *N*-cap and C-terminal Jα-helix conserved among “short” LOV are also shown. α, η, β, and T denote α-helix, 3_{10}-helix, β-strand, and turn, respectively. The percent identities [id. (%)] and similarities [sim. (%)] relative to PmSB-LOV are shown at bottom-right.

Supplementary Figure S1A

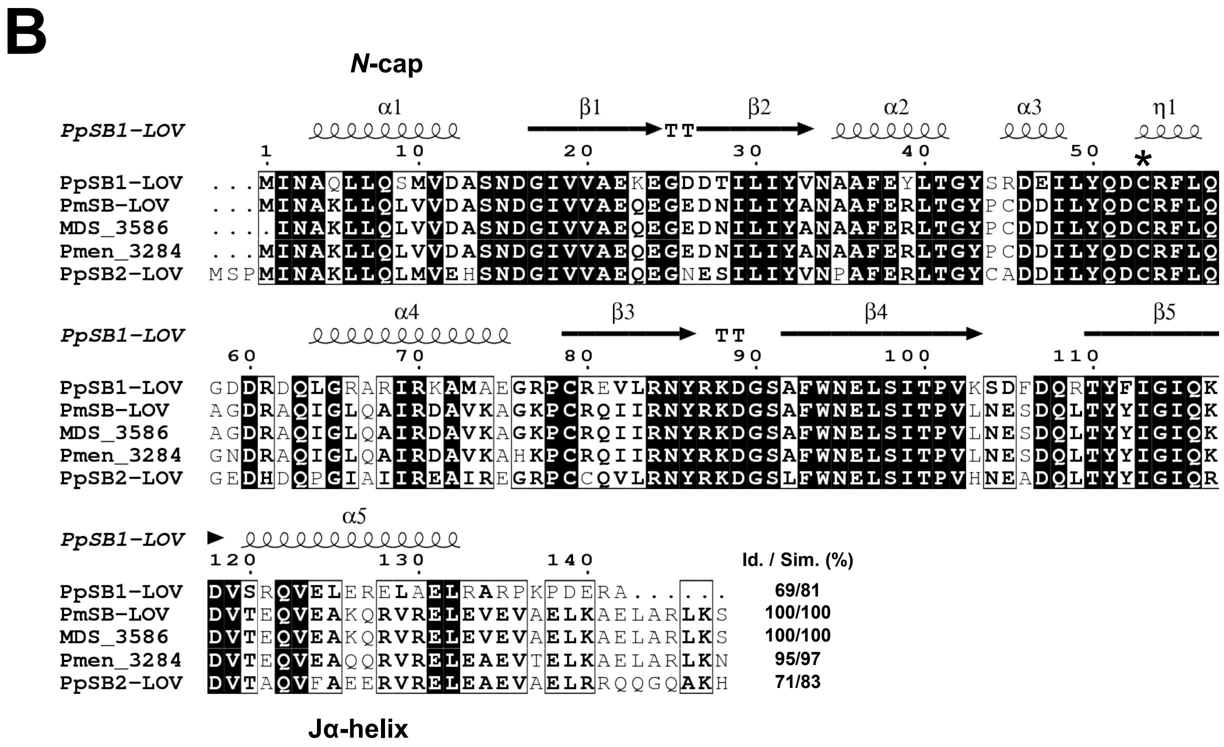

Supplementary Figure S1B

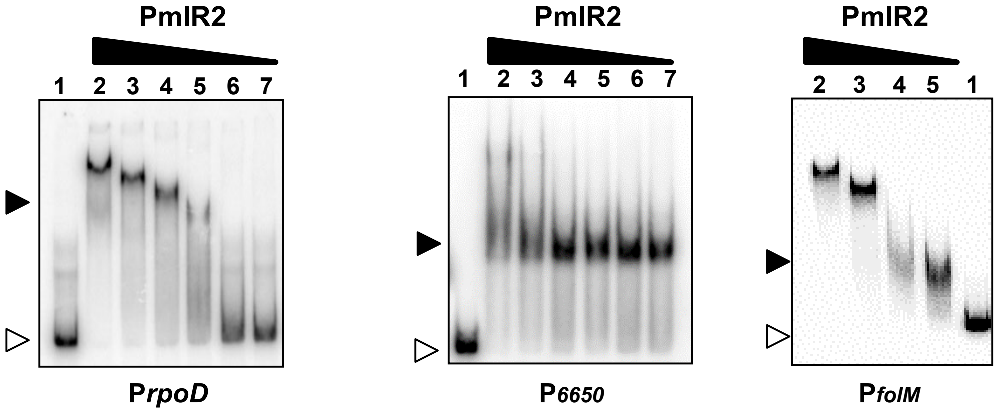

Supplementary Figure S2. Gel-shift assay of the PmlR2 recombinant protein. PmlR2 was added at 160 pmol (lane 2), 80 pmol (lane 3), 40 pmol (lane 4), 20 pmol (lane 5), 10 pmol (lane 6), 5 pmol (lane 7). Lane 1 was a control in which no PmlR2 was present. The probes mixed with PmlR2 were P*rpoD*, P*6650* and P*folM*. Open and closed triangles indicate the probe and protein-DNA complex, respectively. Two independent experiments in technical replicates were performed, and representative data were shown. The original and unprocessed versions of the full length gels were included in Supplementary Figure S9.

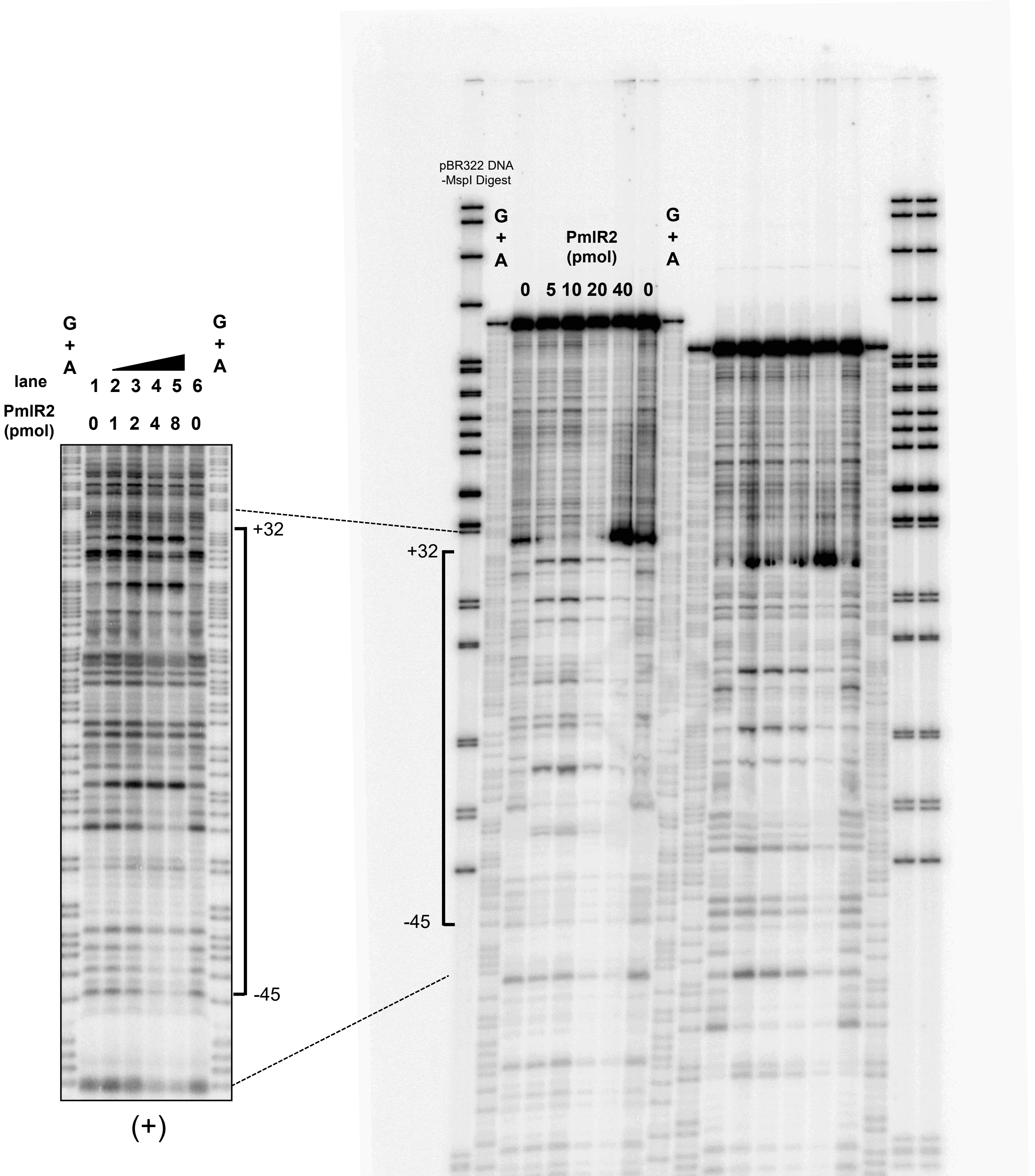

Supplementary Figure S3. Supplementary data for DNase I footprint analysis in the sense (+) strands of P*6650*. The original and unprocessed versions of the full length gel used in this analysis was shown.

Supplementary Figure S3

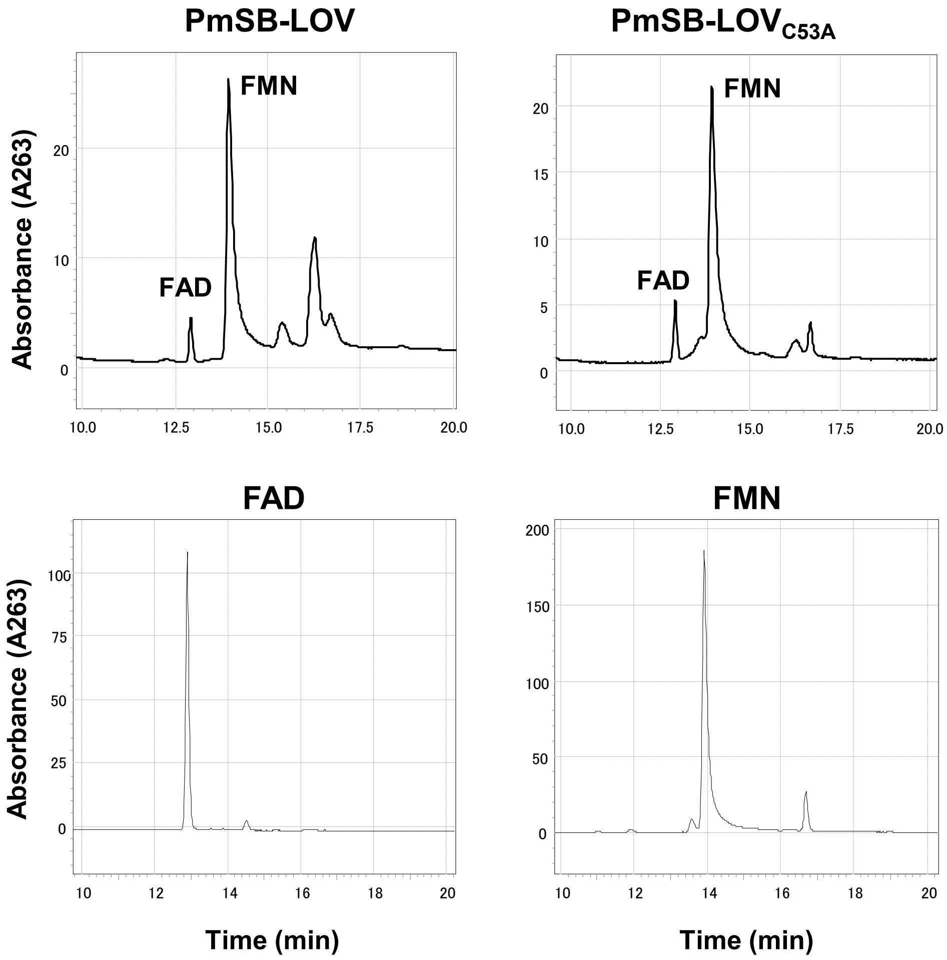

Supplementary Figure S4. HPLC analysis for chromophores bound to PmSB-LOV and PmSB-LOV$_{C53A}$ (upper panels). Commercially available FAD and FMN (lower panels) were used as reference compounds. The chromophores extracted from PmSB-LOV and PmSB-LOV$_{C53A}$ were separated by HPLC. Two independent experiments in technical replicates were performed, and representative data were shown.

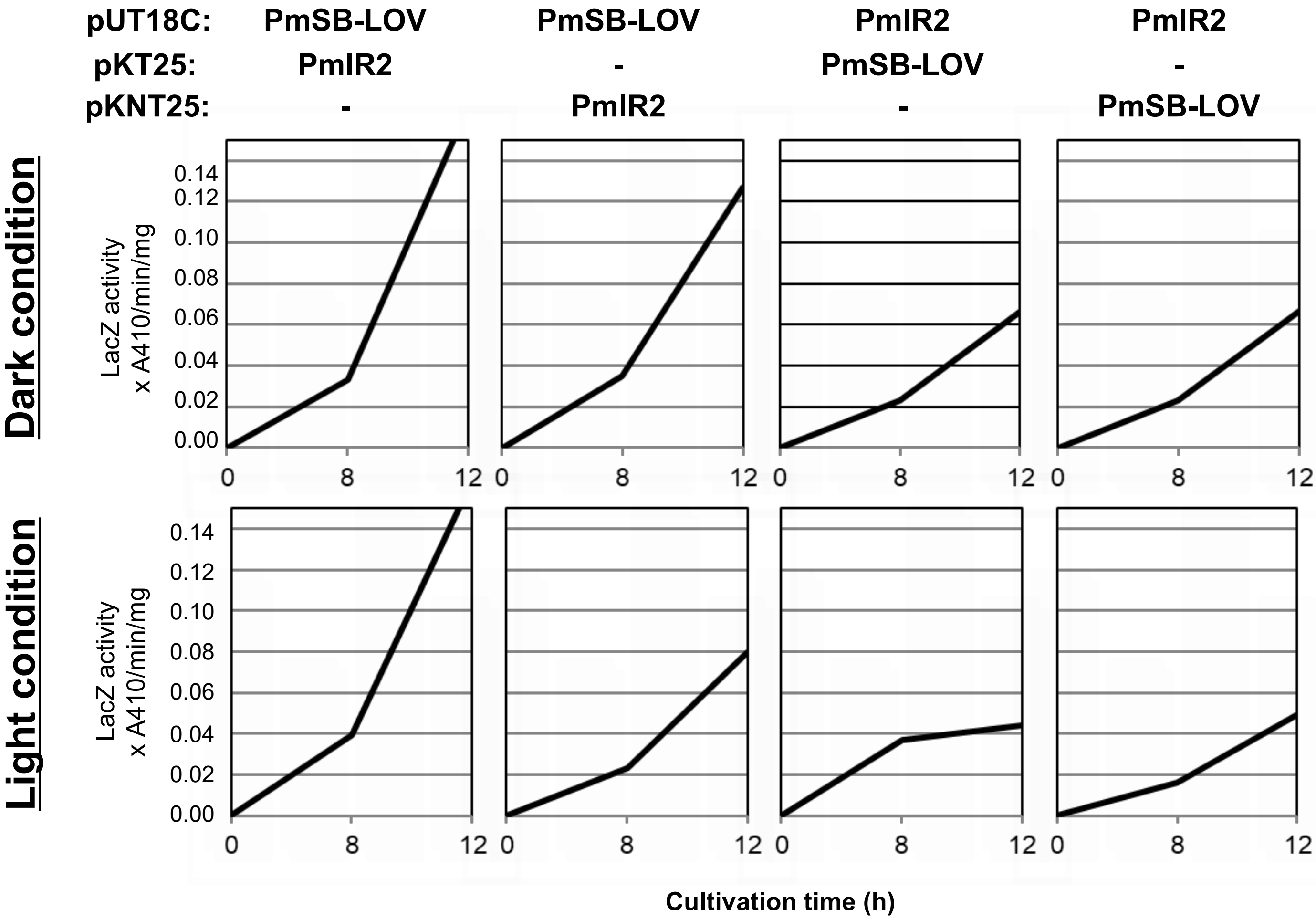

Supplementary Figure S5. Two-hybrid system analysis of PmIR2 and PmSB-LOV in *E. coli*. The *E. coli* BTH101 transformants were cultured under dark and light conditions. In pUT18C, pKT25, and pKNT25, PmIR2 or PmSB-LOV were fused with C-terminus of T18 domain, C-terminus of T25 domain, and N-terminus of T18 domain, respectively. β-galactosidase activity indicating protein-protein interaction was measured at 8 and 12 h. Two independent experiments in biological replicates were performed, and representative data were shown.

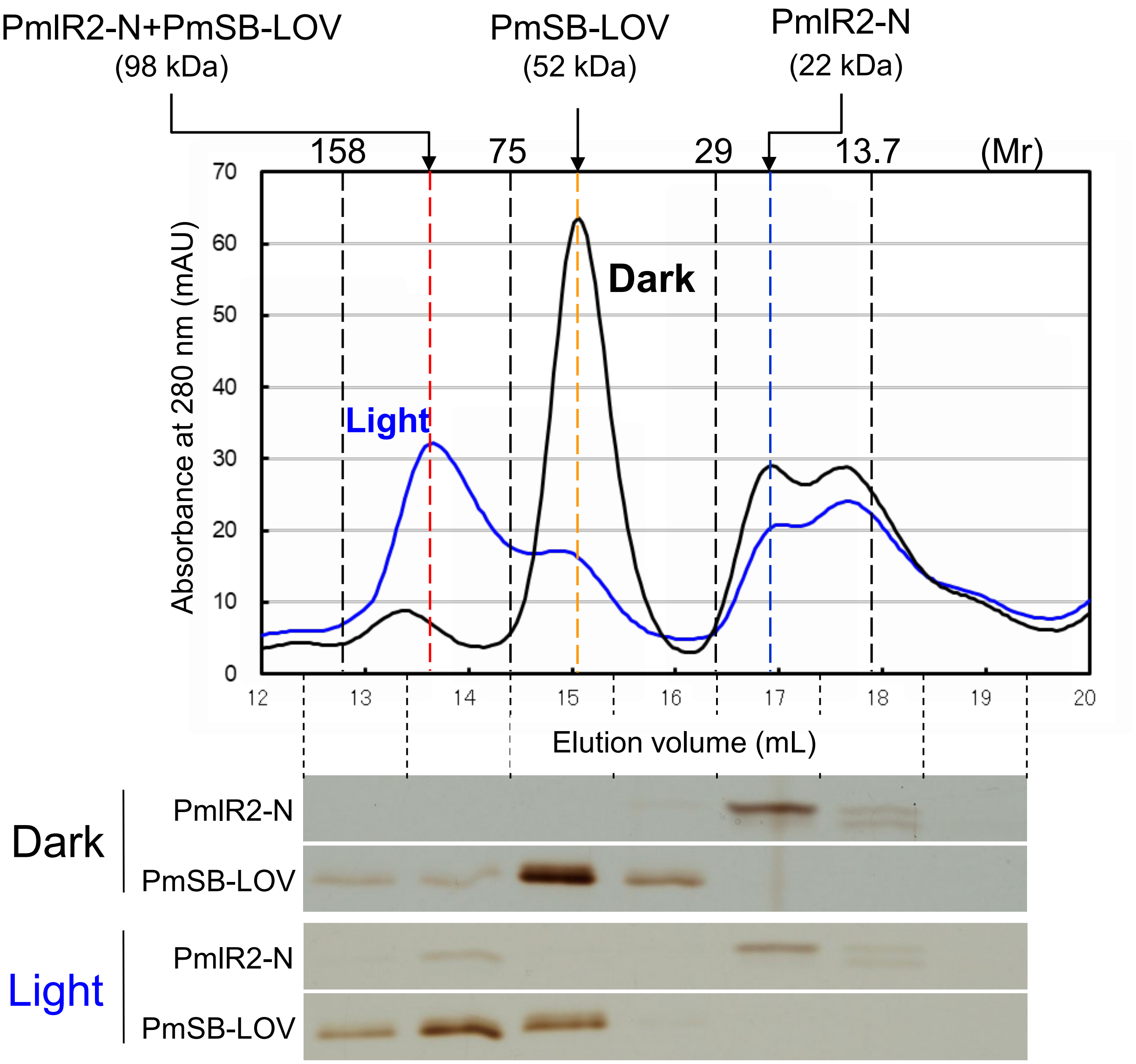

Supplementary Figure S6. Protein-protein interaction between PmIR2-N and PmSB-LOV by gel-filtration chromatography. The recombinant PmIR2-N and PmSB-LOV incubated under dark (solid line) and light (dashed line) conditions were applied to a Superdex 200 HR 10/300 GL column on an ÄKTA FPLC system. PmIR2-N (50 μL; 1.0 mg/mL) and PmSB-LOV (50 μL; 1.0 mg/mL) recombinant proteins were used. The column was equilibrated and developed with 1× PBS at a flow rate of 0.5 mL/min. Molecular size standards (Aldolase , Conalbumin, Carbonic anhydrase, Ribonuclease A indicating 158, 75, 29, and 13.7 kDa, respectively). White light was applied at approximately 50 μmol s^{-1} m^{-2} for 5 min. SDS-PAGE and silver staining were shown at the bottom. Two independent experiments in technical replicates were performed, and representative data were shown. The original and unprocessed versions of the full length gels were included in Supplementary Figure S16.

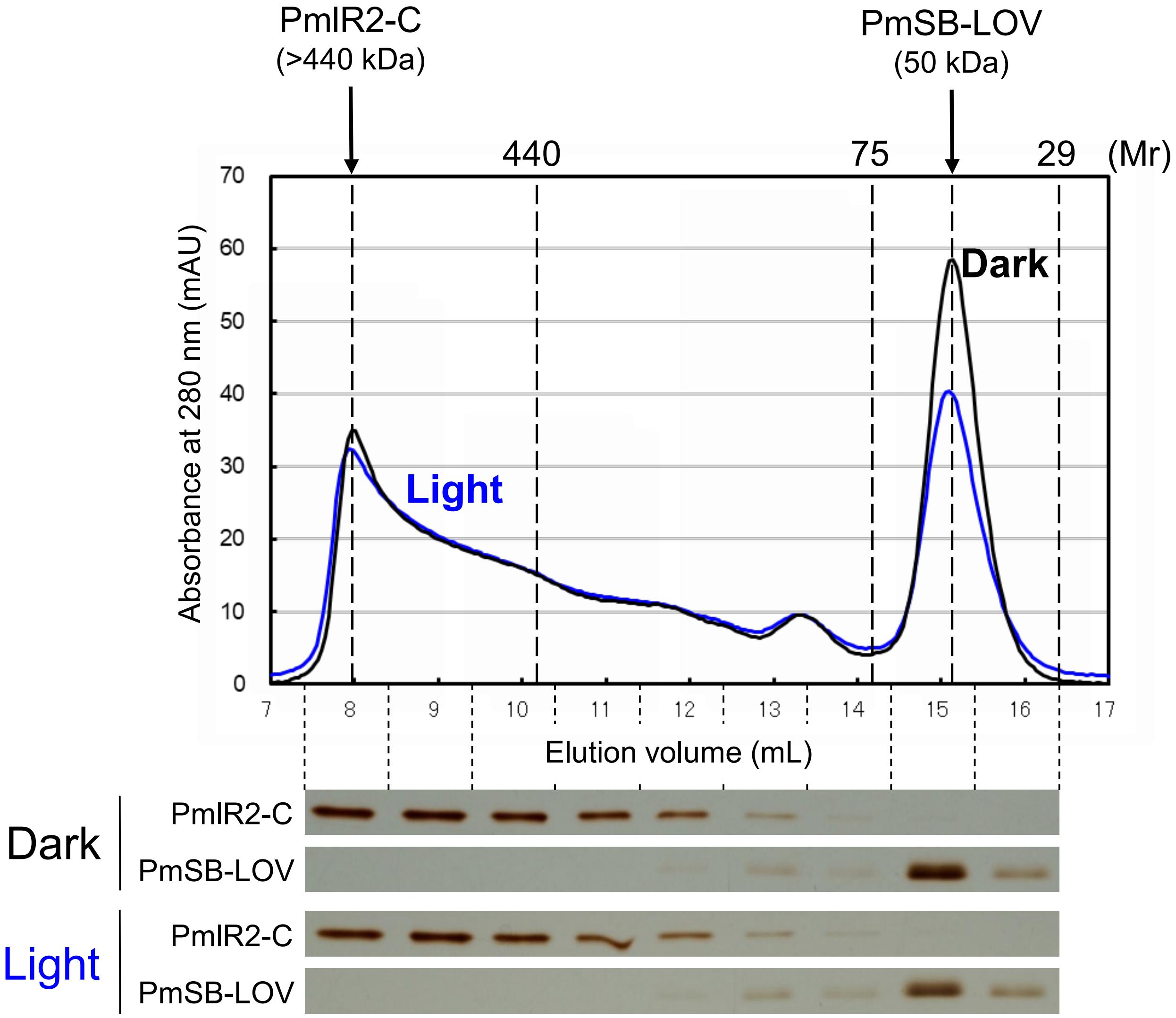

Supplementary Figure S7. Protein-protein interaction between PmIR2-C and PmSB-LOV by gel-filtration chromatography. The recombinant PmIR2-C and PmSB-LOV incubated under dark (solid line) and light (dashed line) conditions were applied to a Superdex 200 HR 10/300 GL column on an ÄKTA FPLC system. PmIR2-C (50 μL; 1.0 mg/mL) and PmSB-LOV (50 μL; 1.0 mg/mL) recombinant proteins were used. The column was equilibrated and developed with 1 × PBS at a flow rate of 0.5 mL/min. Molecular size standards (Ferritin, Conalbumin, and Carbonic anhydrase indicating 440, 75, and 29 kDa, respectively). White light was applied at approximately 50 μmol s^{-1} m^{-2} for 5 min. SDS-PAGE and silver staining were shown at the bottom. Two independent experiments in technical replicates were performed, and representative data were shown. The original and unprocessed versions of the full length gels were included in Supplementary Figure S17.

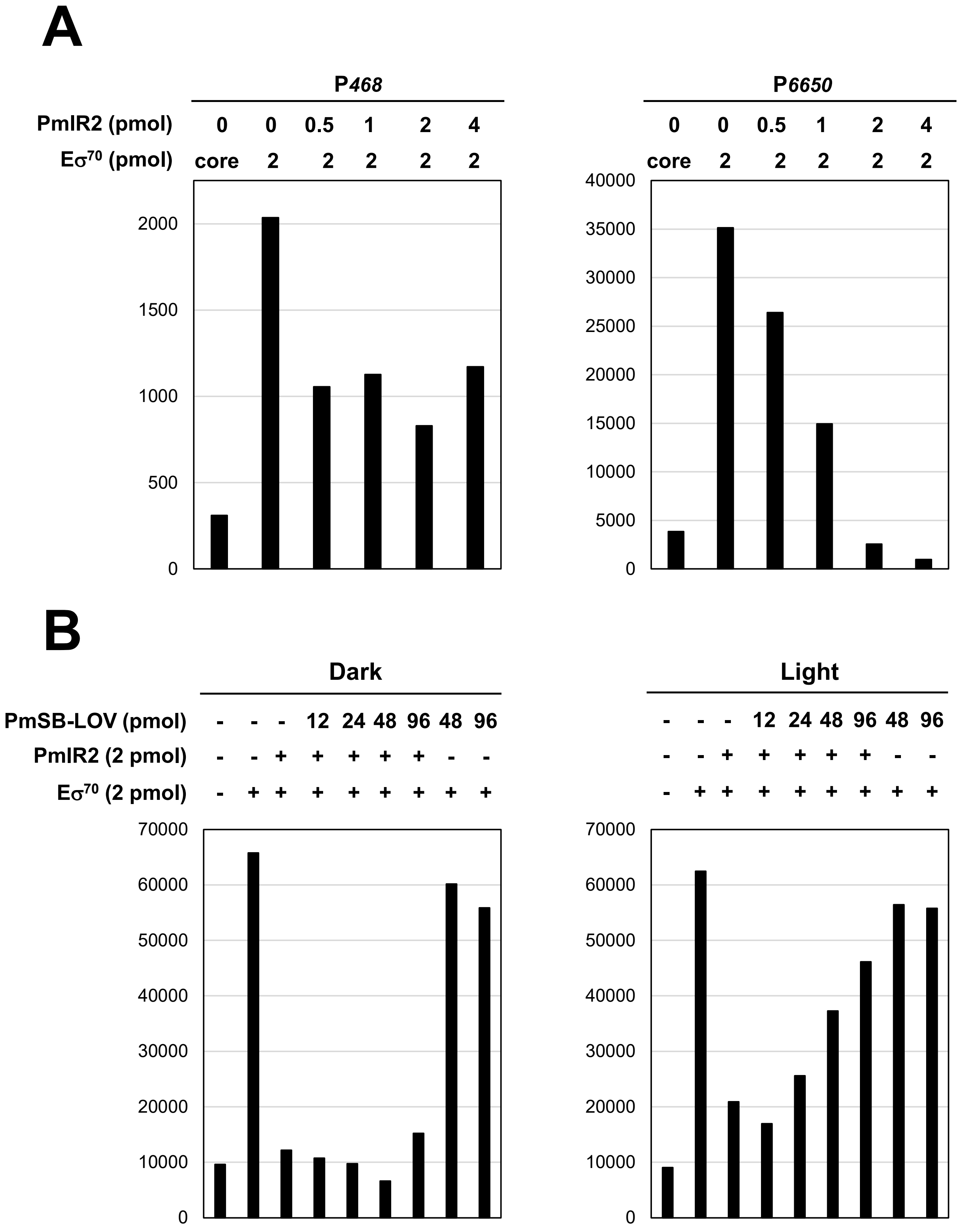

Supplementary Figure S8. Quantification of the intensity of the band signal obtained with *In vitro* transcriptional runoff assay (Fig. 8). ImageQuant TL version 8.1 was used for the signal quantification.

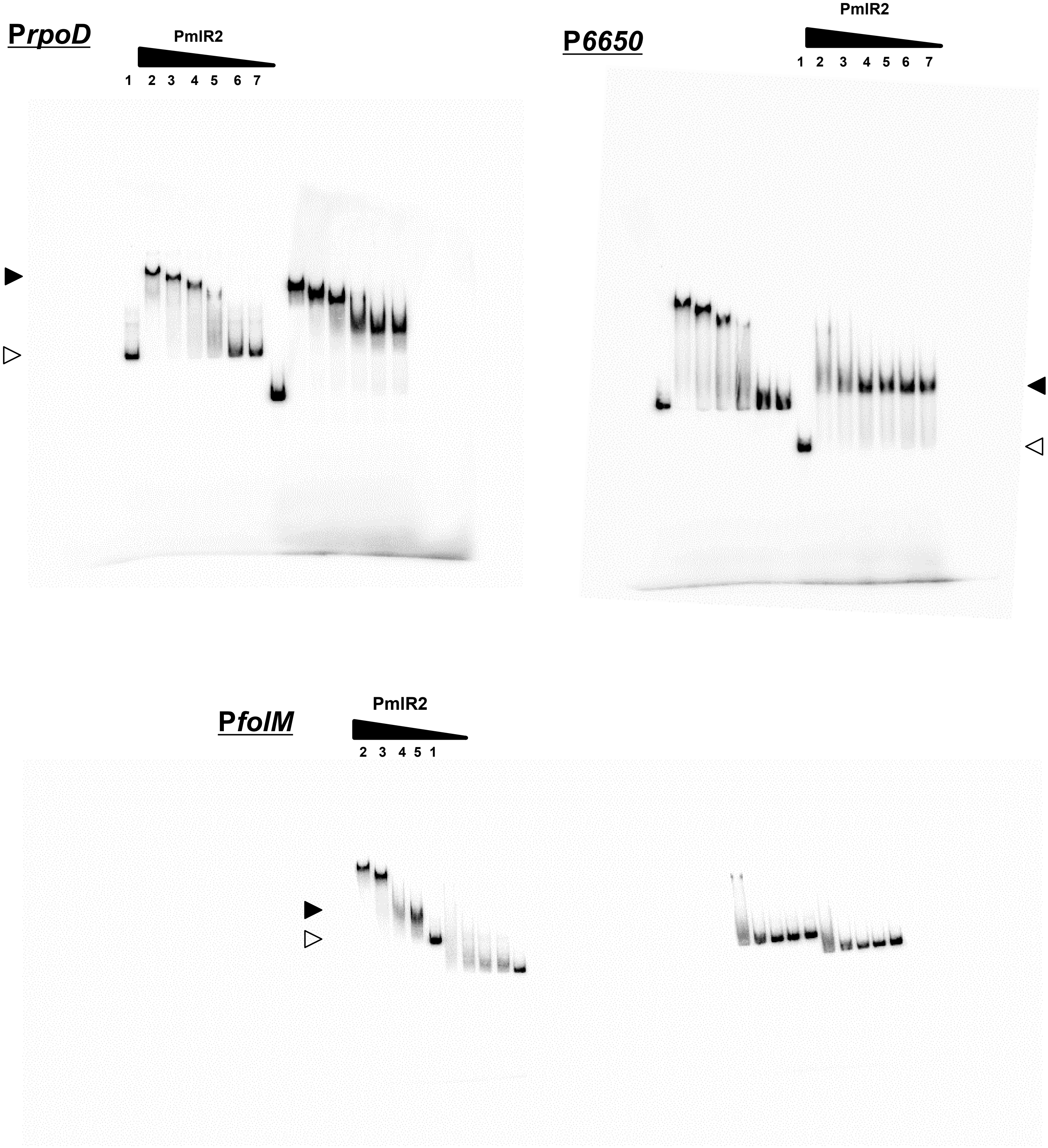

Supplementary Figure S9. The original and unprocessed versions of the full length gels used in Supplementary Figure S2.

Supplementary Figure S9

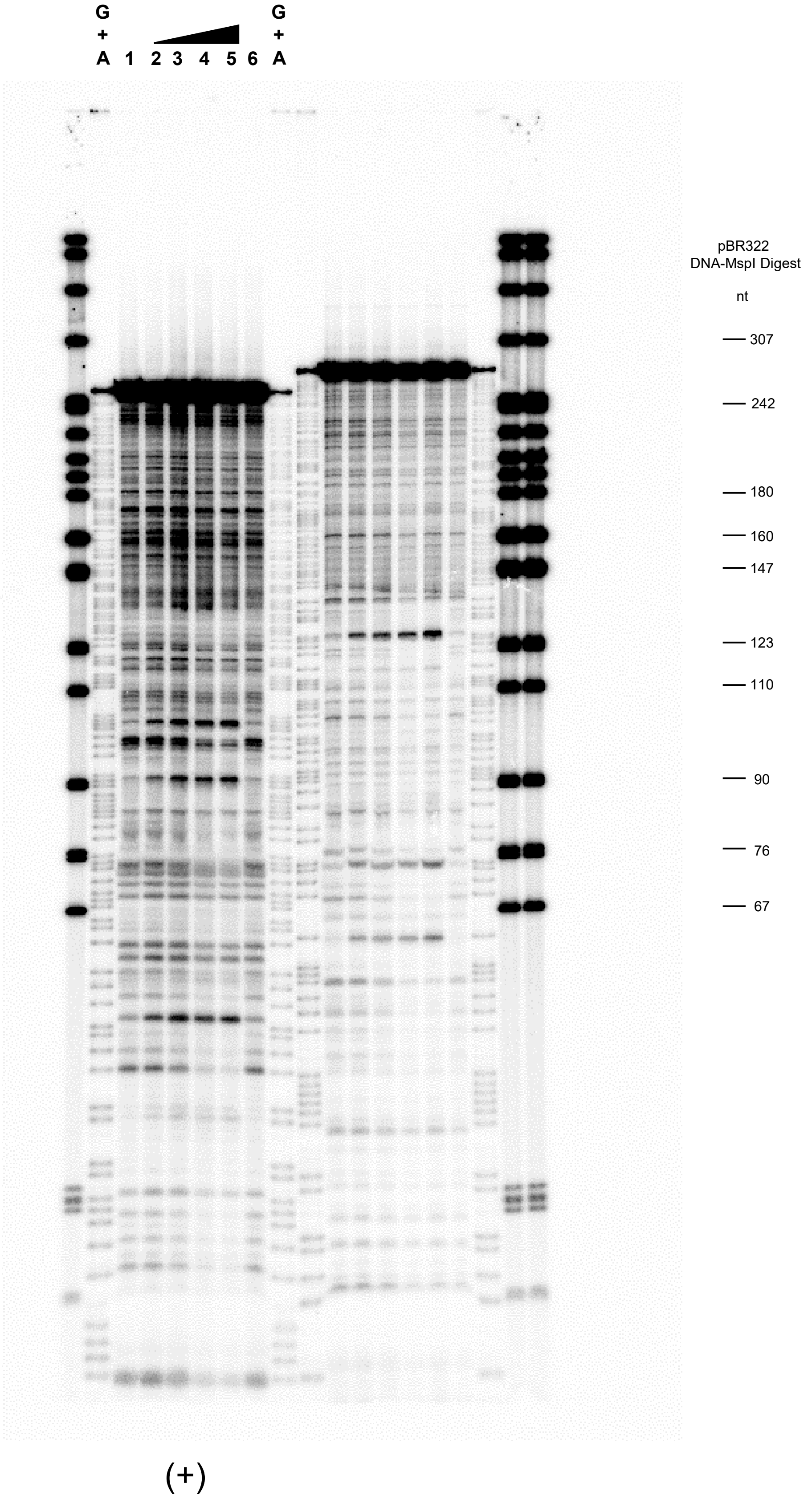

Supplementary Figure S10. The original and unprocessed versions of the full length gels used in Fig. 2B (Left panel)

Supplementary Figure S10

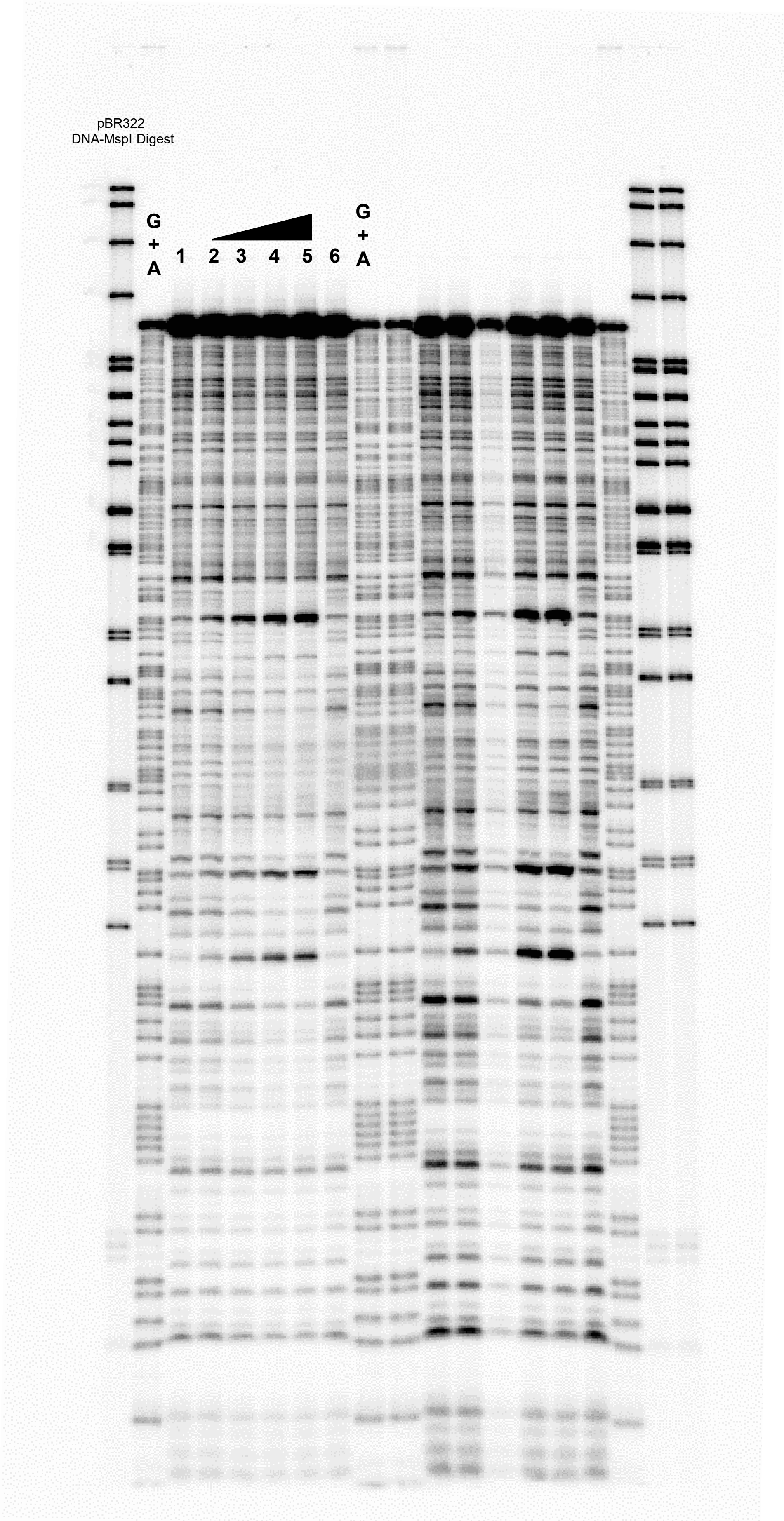

Supplementary Figure S11. The original and unprocessed versions of the full length gels used in Fig. 2B (right panel)

Supplementary Figure S11

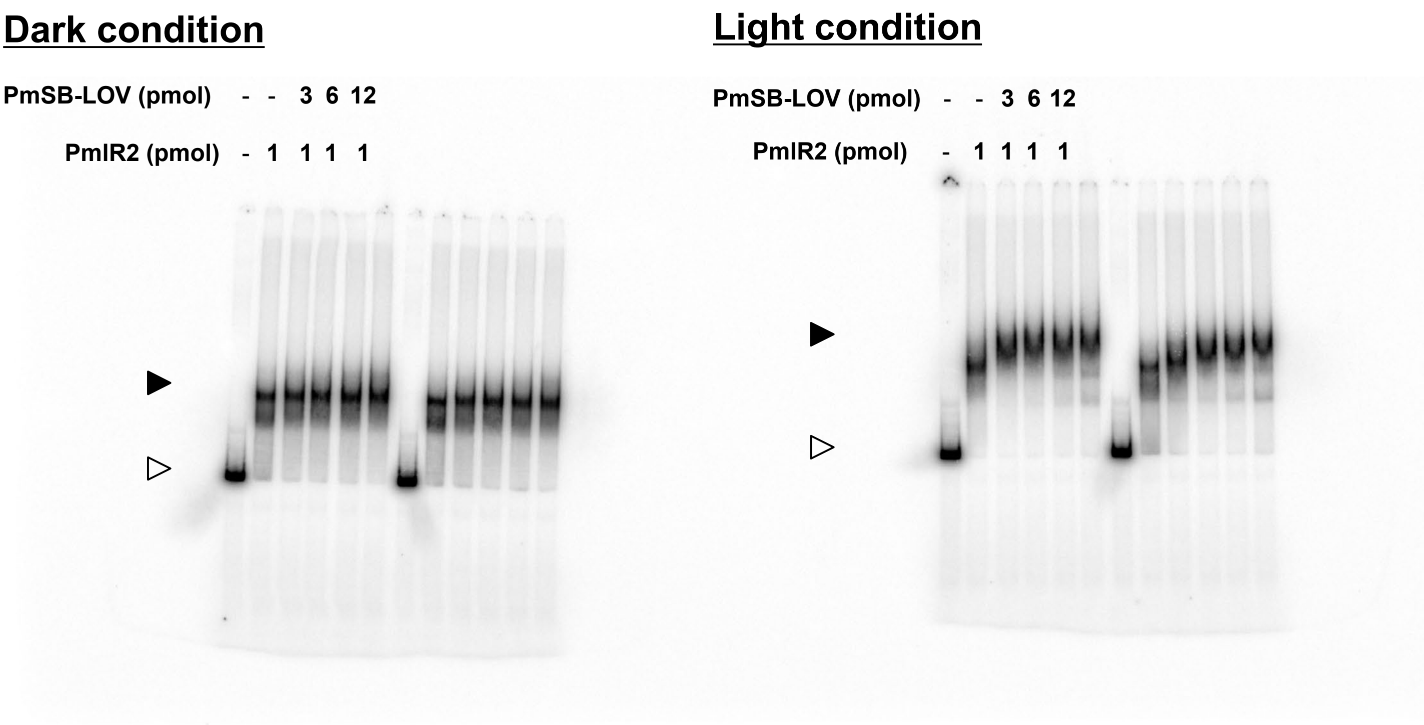

Supplementary Figure S12. The original and unprocessed versions of the full length gels used in Fig. 5

Dark condition

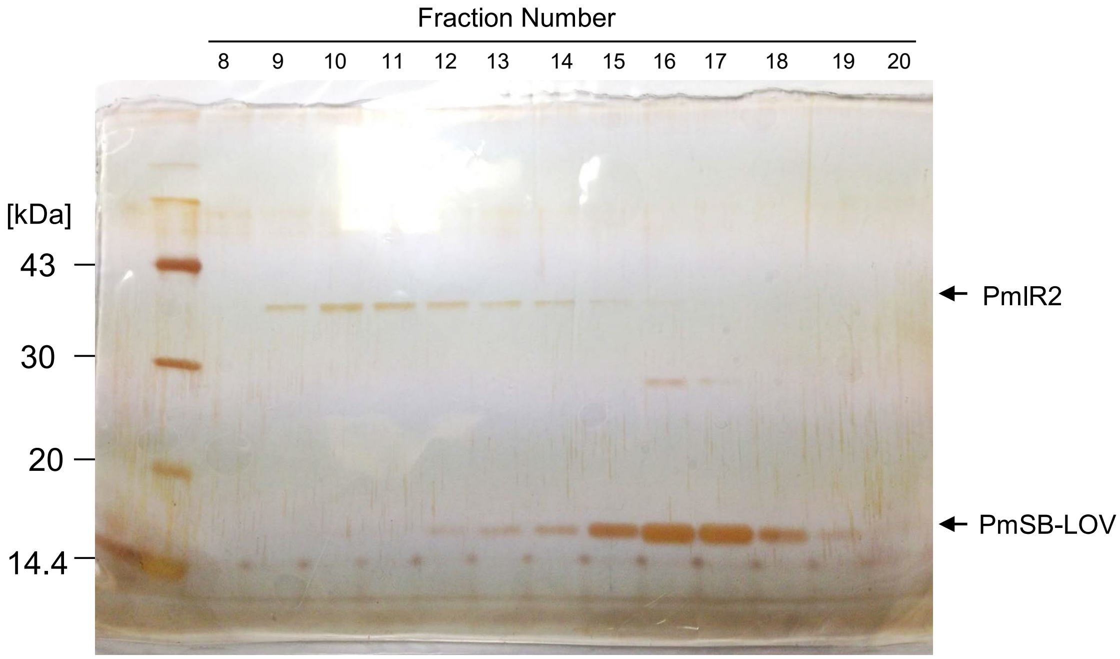

Light condition

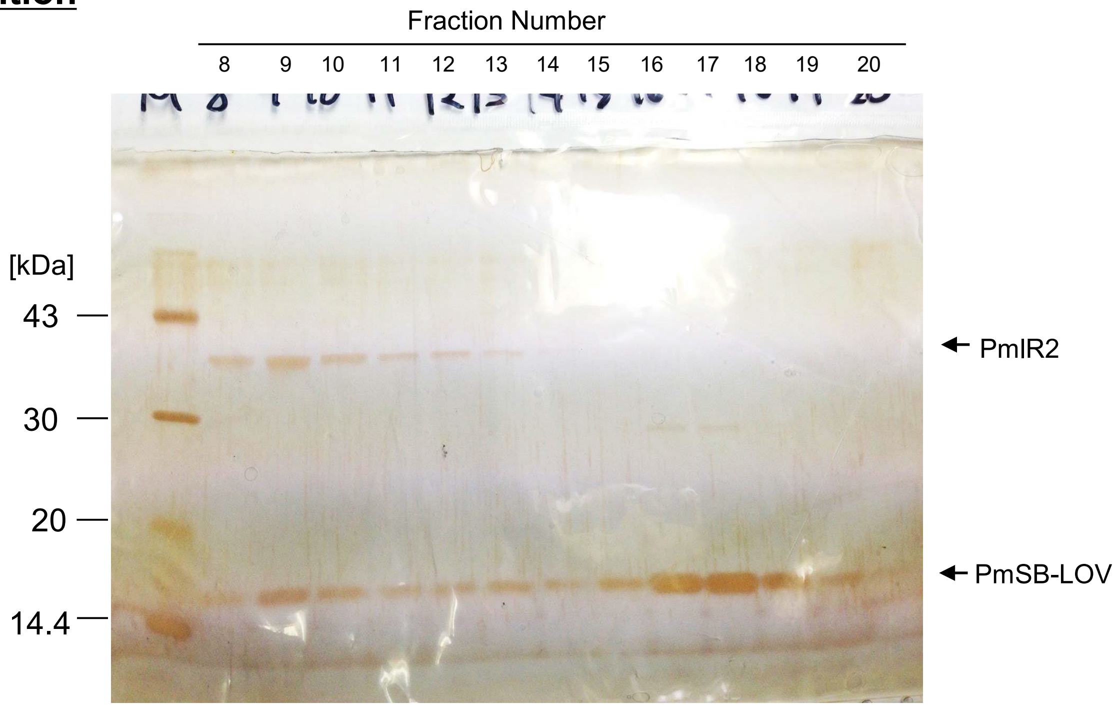

Supplementary Figure S13. The original and unprocessed versions of the full length gels used in Fig. 6

Fig. 8A P*468*

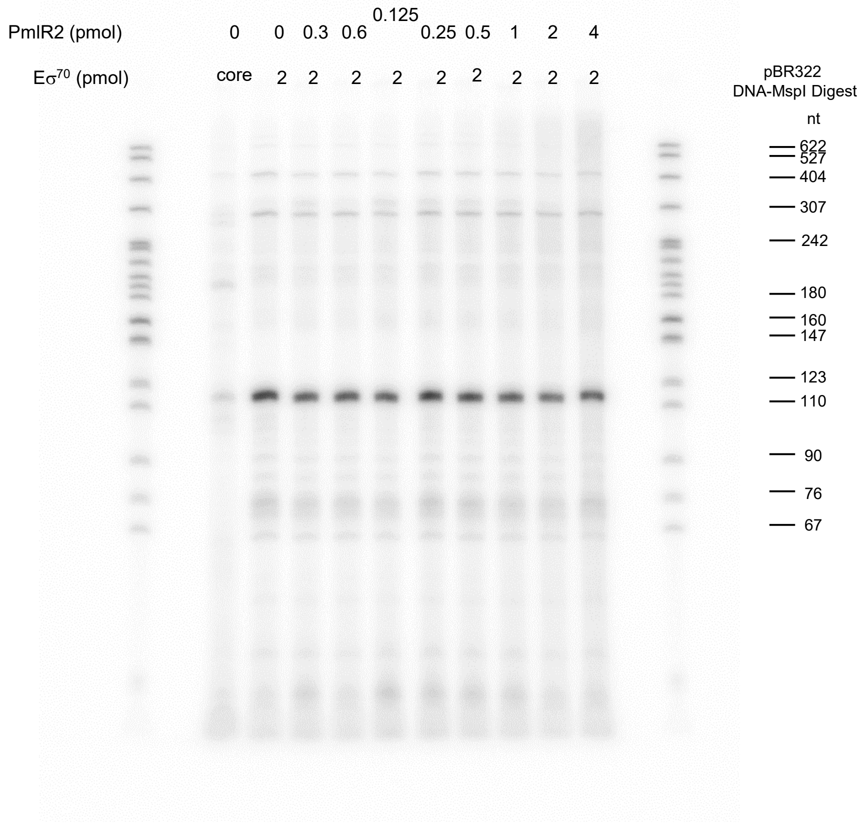

Fig. 8A P*6650*

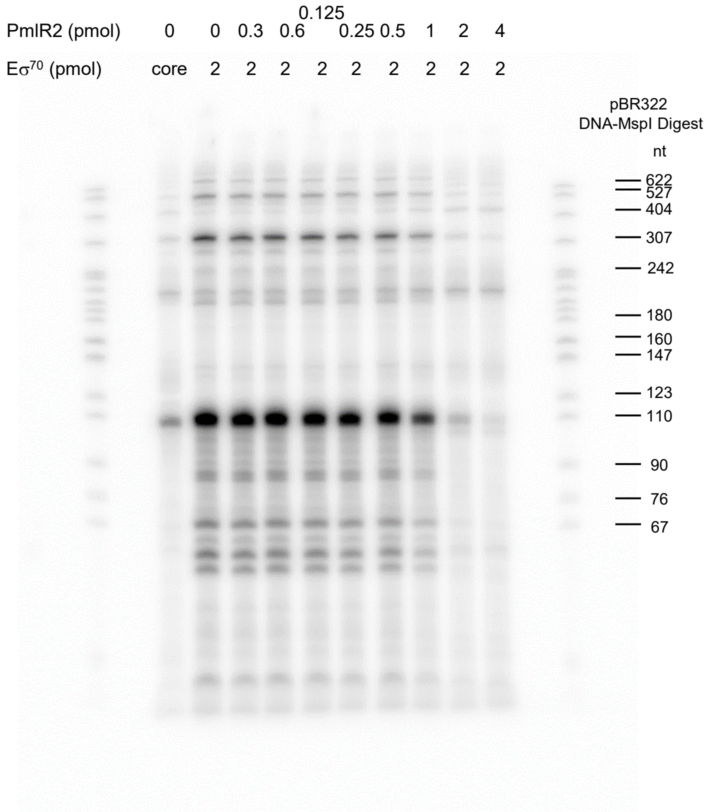

Supplementary Figure S14. The original and unprocessed versions of the full length gels used in Fig. 8A

Fig. 8B Dark condition

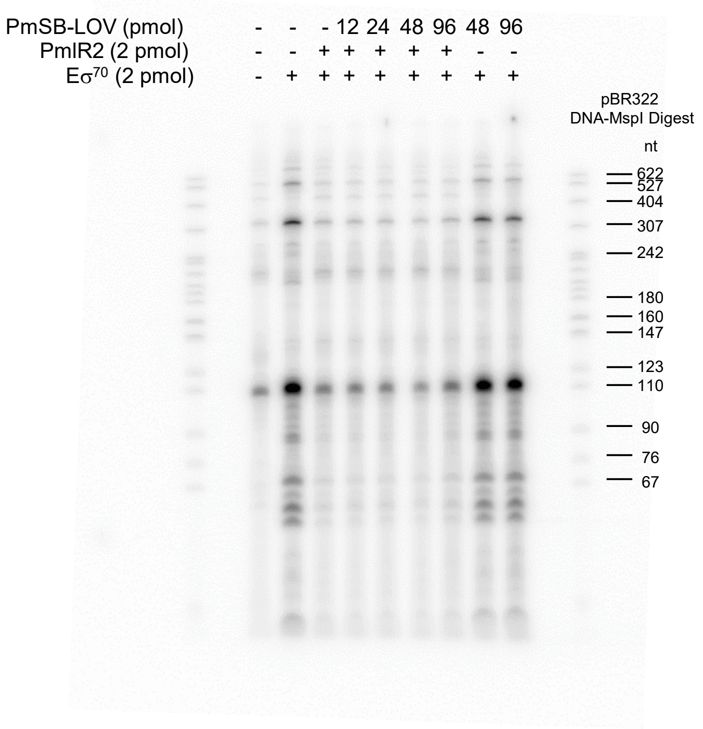

Fig. 8B Light condition

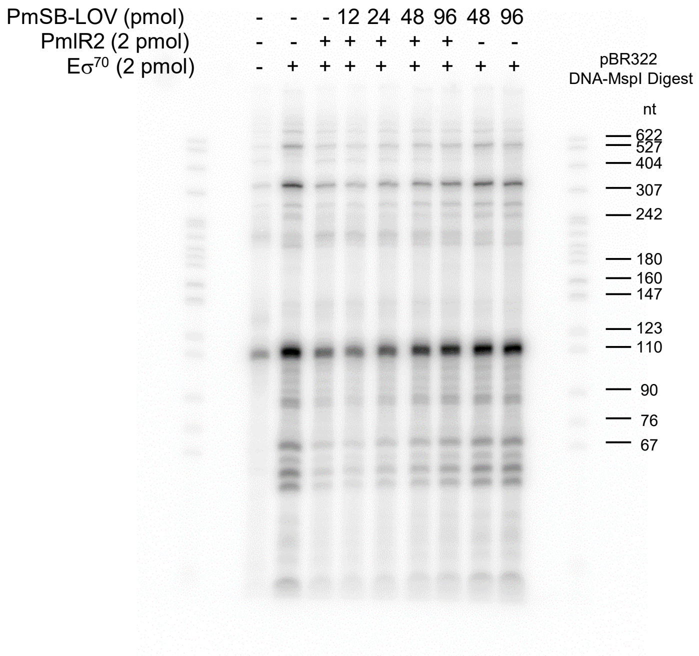

Supplementary Figure S15. The original and unprocessed versions of the full length gels used in Fig. 8B

Dark condition

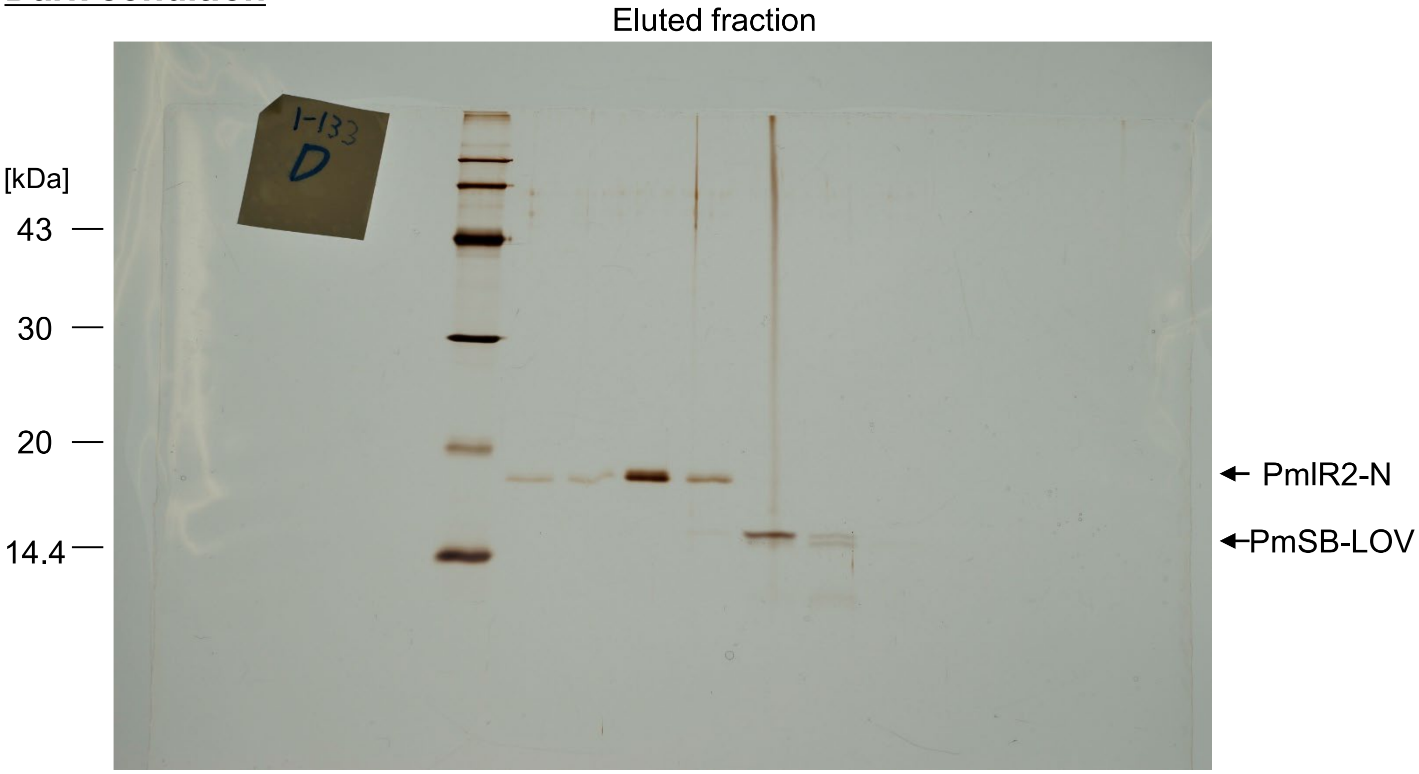

Light condition

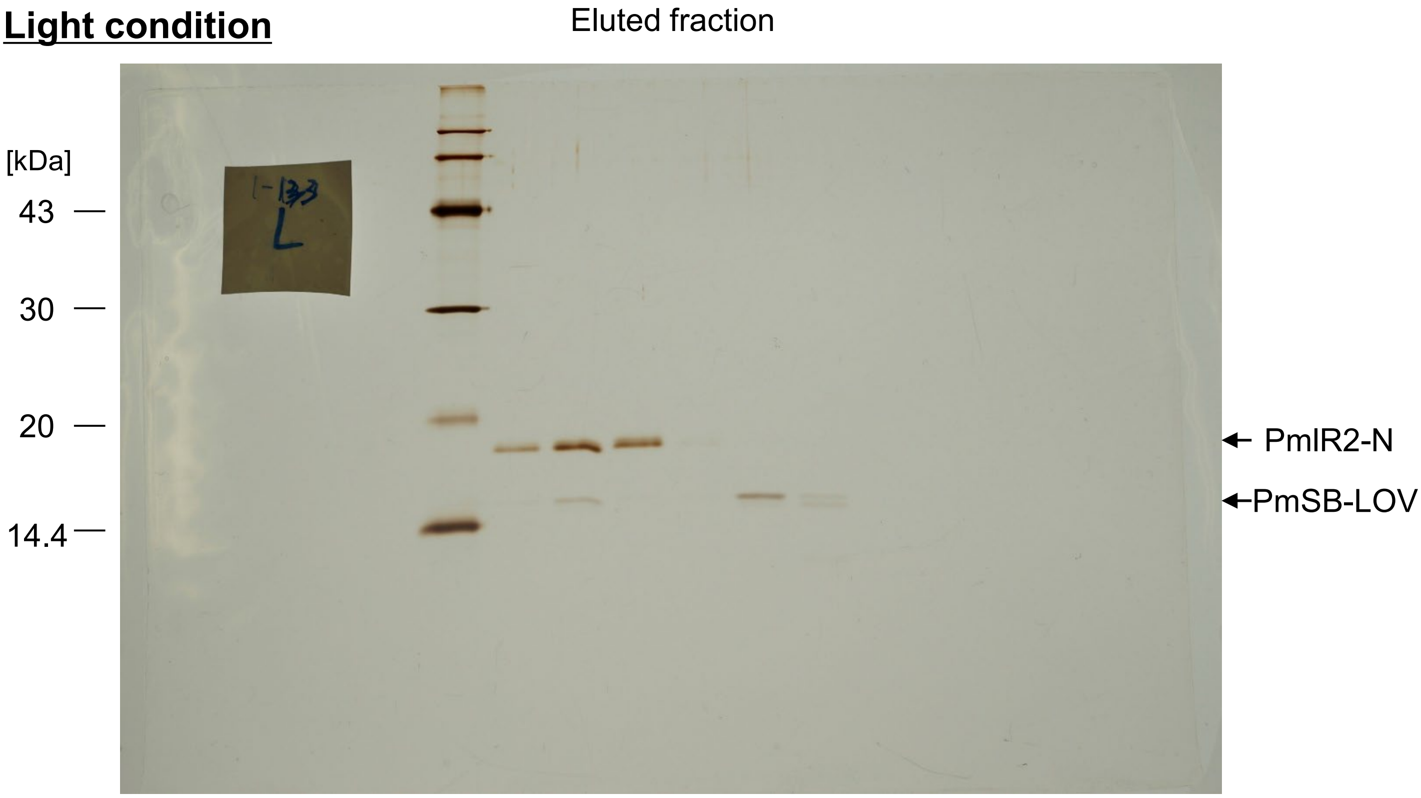

Supplementary Figure S16. The original and unprocessed versions of the full length gels used in Supplementary Figure S6.

Supplementary Figure S16

Dark condition

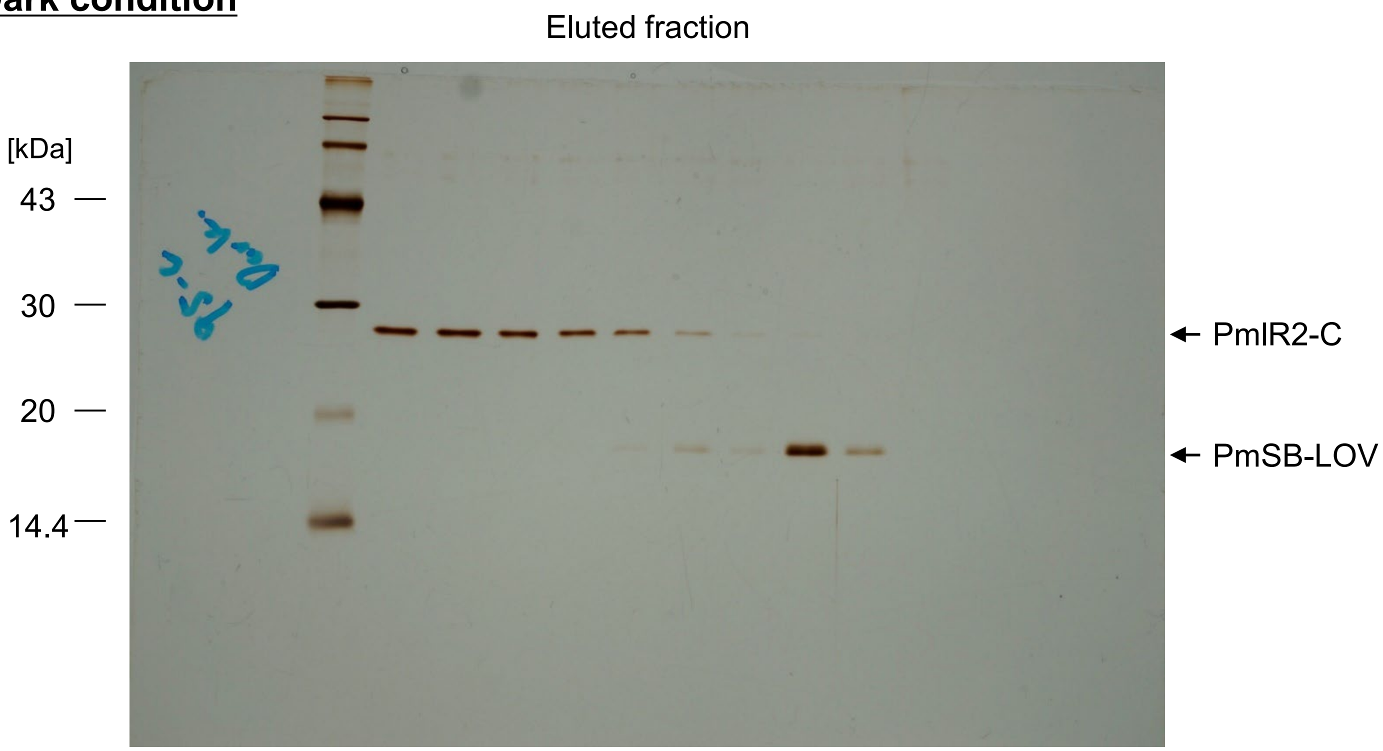

Light condition

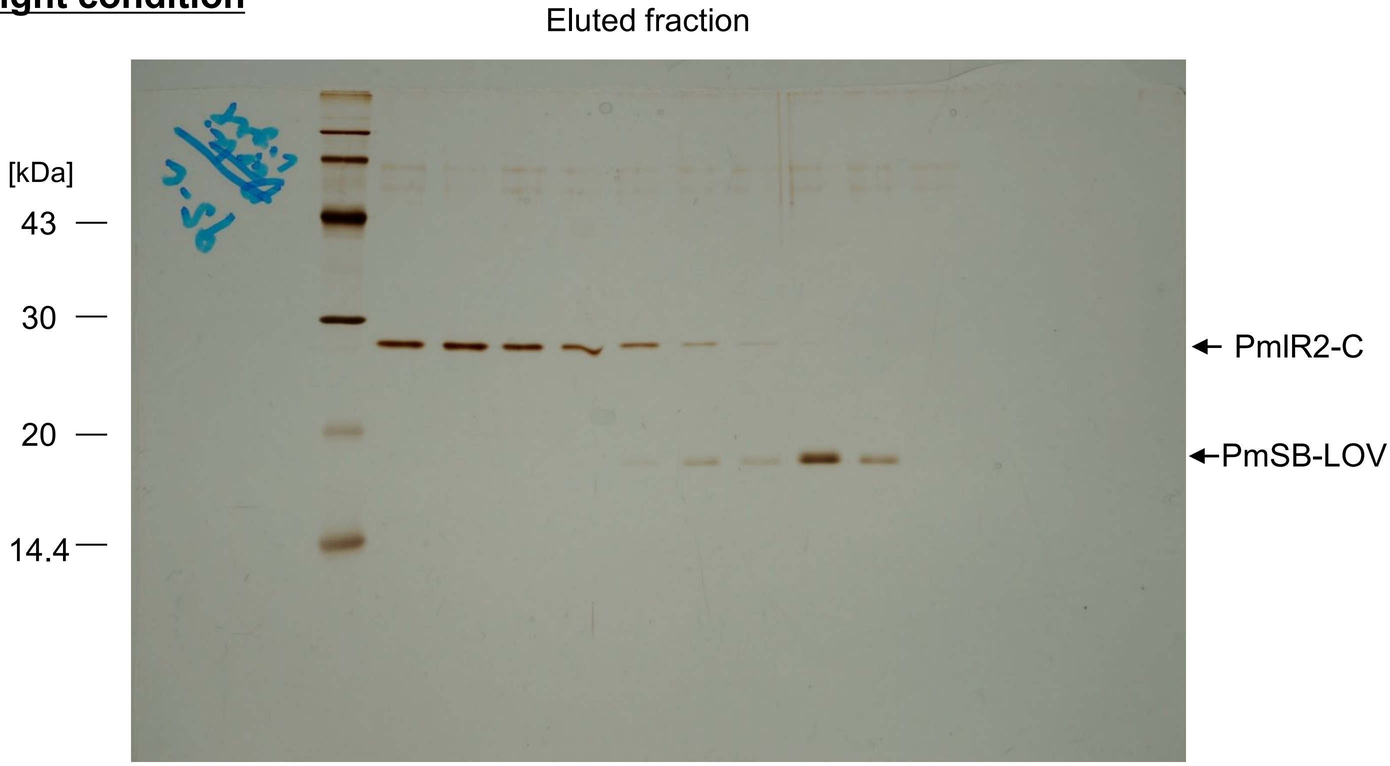

Supplementary Figure S17. The original and unprocessed versions of the full length gels used in Supplementary Figure S7.

Supplementary Materials
Reference

(1) Larkin MA, Blackshields G, Brown NP, Chenna R, McGettigan PA, McWilliam H, Valentin F, Wallace IM, Wilm A, Lopez R, Thompson JD, Gibson TJ, Higgins DG. Clustal W and Clustal X version 2.0. *Bioinformatics*. 2007, 23(21):2947-2948.
(2) Gouet P, Robert X, Courcelle E. ESPript/ENDscript: Extracting and rendering sequence and 3D information from atomic structures of proteins. *Nucleic Acids Res*. 2003, 31(13):3320-3323.
(3) Robert X, Gouet P. Deciphering key features in protein structures with the new ENDscript server. *Nucleic Acids Res*. 2014, 42(Web Server issue):W320-324.
